# Agentic AI-enhanced digital twins for Smart City civil infrastructure: A secure, autonomous and auditable management framework

**DOI:** 10.1371/journal.pone.0353610

**Published:** 2026-07-17

**Authors:** Toqeer Ali Syed, Ali Akarma, Ali Alatify, Muhammad Tayyab Naqash, Abdulaziz Alqurashi

**Affiliations:** 1 AI Center, Faculty of Computer and Information Systems, Islamic University of Madinah, Madinah, Saudi Arabia; 2 AI V&V Lab, King Fahd University of Petroleum and Minerals, Dhahran, Saudi Arabia; 3 Department of Civil Engineering, Prince Sattam Bin Abdulaziz University, Al-Kharj, Saudi Arabia; 4 Civil Engineering Department, Faculty of Engineering, Islamic University of Madinah, Madinah, Saudi Arabia; King Abdulaziz University, SAUDI ARABIA

## Abstract

Smart city implementation increasingly relies on sensing and analytics; however, a persistent operational gap remains between anomaly detection and safe, timely, and accountable intervention in civil infrastructure systems. This paper proposes an Agentic AI-supported Digital Twin framework for smart city civil infrastructure management, where monitoring and action are linked and auditability is maintained. The Digital Twin continuously updates asset and network models of bridges, roads, and water infrastructure using multi-stream telemetry, incorporating state estimation, predictive maintenance, and what-if simulation services. At the orchestration layer, an agent-based Perception–Conceptualization–Action workflow implemented with LangChain and LangGraph enables cross-domain reasoning and coordinated mitigation planning through controlled API calls to municipal data. A permissioned blockchain cryptographically binds observations, approvals, and executed interventions, ensuring provenance, governance, and tamper evidence. To evaluate the framework, 18,000 incident simulations were conducted across five architectural configurations and three scenario complexity levels over 30 independent runs. This simulation study characterises framework behaviour under controlled stochastic conditions and does not constitute real-world operational validation. Ablation analysis isolates each component’s contribution, demonstrating that latency and mitigation gains are primarily attributable to multi-agent orchestration, while the blockchain layer drives decision auditability. Across all configurations, the fully agentic system substantially outperforms the rule-based baseline: mean detection latency of 3,197 s vs. 39,374 s, mitigation success rate of 66.2% vs. 45.5%, blockchain-anchored decision justification of 71.8% vs. 0%, and operator workload reduction of 91.7% vs. 0%. These results demonstrate that combining simulation-enabled digital twins with governance-aware agentic orchestration measurably improves response efficiency, recommendation quality, and action accountability within the bounds of a synthetic evaluation environment.

## 1. Introduction

The high rate of urbanization and the growth of urban metropolitan areas have put unprecedented strains on civil infrastructure systems, including bridges, road systems, water systems, drainage systems, and other public works property [1]. These systems shape the city and are directly linked to the safety of the population, to economic sustainability, and to livelihood [2]. Traditional reactive and cycle-based maintenance teams have become less effective due to ageing infrastructure, extra traffic pressures, extreme climatic events and lack of resources for maintenance. The management of modern cities needs data-driven, yet predictive, adaptive and actionable management strategies that can help in timely and well-supported interventions in the cities [3].

**Fig 1:**
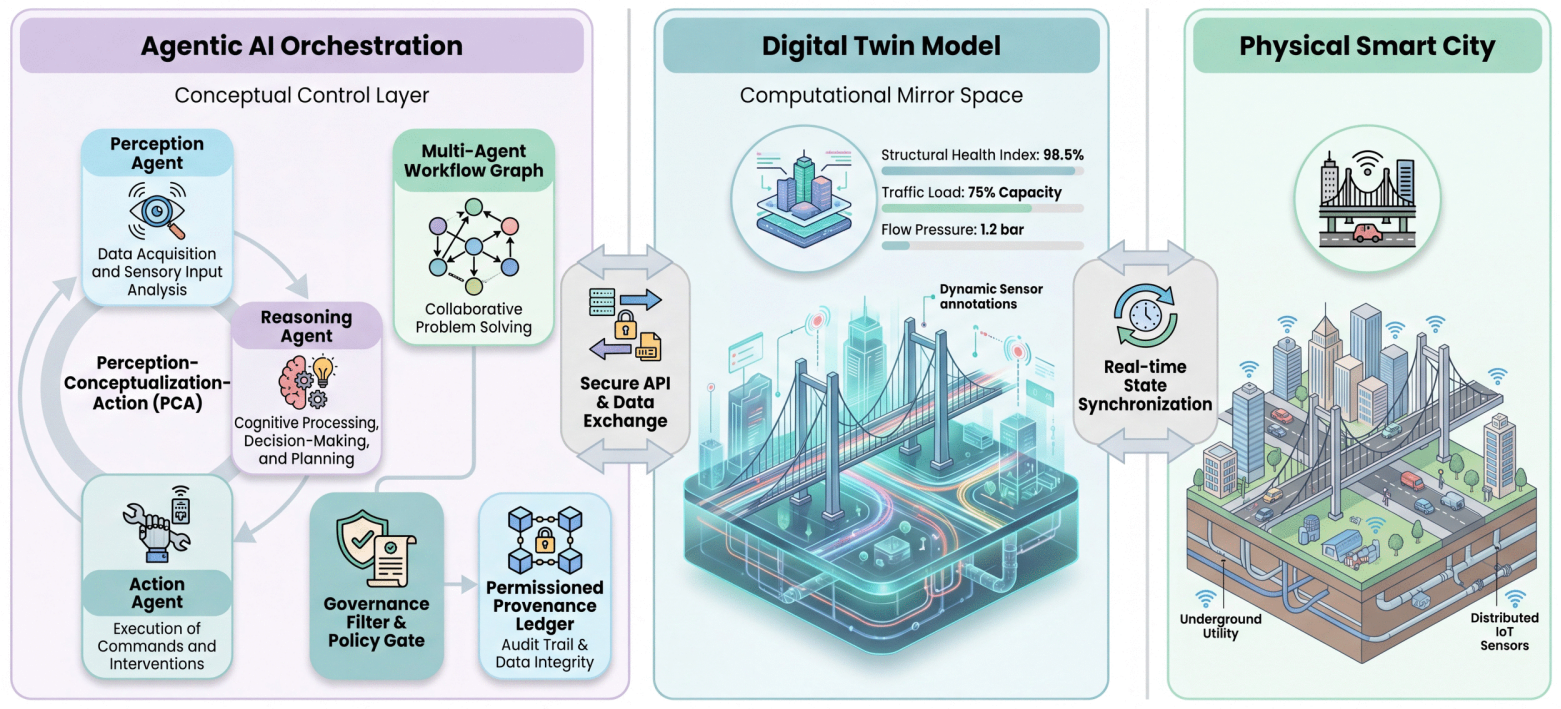
Proposed Agentic AI–Enhanced Digital Twin architecture showing multi-agent orchestration, computational mirror space, and synchronised physical smart city infrastructure with blockchain-backed auditability.

The idea of smart city has been introduced as a matter of smart technology to improve the efficiency and resilience of the city’s operations. However, at present, most of the smart city projects address high level services, dashboards or individual optimization tasks, and management of civil infrastructure is still fragmented and mostly relies on experts’ opinion [4]. Such systems are available but there is a gap in seamlessly transforming signals into coordinated, safe and auditable actions across departments within municipal offices. This gap will need to be bridged by only making connections between: sensing, modelling, reasoning, and execution of workflows and actions more explicit and accountable – as shown in [5]. The proposed Agentic AI–Enhanced Digital Twin framework includes multi-agent orchestration, a computational space mirroring the physical space, and the SMART city infrastructure, as shown in (Fig 1)).

Digital Twin (DT) technology is a promising solution to tackle such challenges. A civil infrastructure digital twin is a continuously updated synthetic model of the physical infrastructure, which integrates structural, hydraulic and traffic-related data as a single model of the city’s engineered systems [6]. Unlike static information models, a DT is dynamic and can be used to evaluate interventions via simulation before implementation in the physical world. This feature is especially important for situations where the degradation is safety critical, like bridges or trunk pipelines, where the speed of identifying degradation and the evaluation of mitigation measures is significant, and can make an impact in reducing safety risk [7].

The majority of the digital twin applications to cities are still descriptive or analytic. They enable monitoring, visualization, and scenario analysis even when offline, and rely on humans to interpret the alerts, formulate response strategies, and coordinate between departments [8]. This manual synthesis process becomes an impediment when the infrastructure networks become more interdependent with each other especially for time-sensitive scenario [9]. This requires development of digital twins being supported by autonomous decision-making systems within stringent safety and governance requirements.

Agentic AI is the logical next step in the digital twin concept. The agentic AI system is designed to follow a Perception–Conceptualization–Action (PCA) cycle, where it processes structured inputs, generates conceptualizations and predictions about future events, and takes actions to achieve the defined goals [10]. Agentic AI can advance in handling alerts to structured alert triage, cross-asset reasoning, and coordinated intervention planning, in combination with a civil infrastructure digital twin. However, with recent advances in tool using large language models and orchestration frameworks like LangChain, the concept of multi-agent systems is now become conceptually feasible, where specialized agents query models, invoke simulations and call external systems through well-defined APIs [11]. However, for safety-critical civil infrastructure, using this agentic autonomy necessitates a careful design of the system enabling transparency, controllability and traceability.

Governance and trust represent a further challenge. Decision-making should be explainable and auditable for public infrastructure, not just in compliance with regulation, but for post incident analysis, whether the decisions are made by the public infrastructure or semi-autonomously. In this context, blockchain technology is a complementary capability, since it provides an immutable and decentralized ledger to capture the provenance of decisions [12]. Observations, reasoning artefacts, human approvals and actions taken by agentic systems can be committed to a blockchain ledger to ensure that everything that happens is verifiable and impossible to tamper with without revealing sensitive raw data [13].

In this paper, a conceptual and simulation evaluated framework using Agentic AI with the Digital Twin specific to smart city civil infrastructure management is presented. The framework consists of a digital twin simulation layer, auditability by a permissioned blockchain, and a multi-agent PCA layer, inspired by LangChain and LangGraph. Unlike the general smart city solutions, the proposed framework is specifically targeted towards the civil infrastructure assets such as bridges, roads and water systems, and the operations that take place in these systems to monitor, assess and intervene within them. Evaluated via a large-scale synthetic Monte Carlo simulation study with 18,000 incident simulations for five architectural configurations, and three complexity of scenarios. Most importantly, the evaluation is presented as a characterization study of the comparative simulated behaviour of the framework and the results are limited to synthetic environment; the direction of real-world operational validation is identified as a future research direction.

The main contributions of this work are:

- A unified agentic digital twin architectural framework with blockchain-anchored provenance.
- A controlled ablation study isolating the marginal contribution of individual architectural components (digital twin, single-agent, multi-agent orchestration, and blockchain).
- A run-level statistical analysis, treating each of 30 independent simulation runs as the primary experimental unit, addressing nested data structure concerns.
- Empirical evidence from 18,000 simulated incidents demonstrating that multi-agent orchestration is the primary driver of latency and success improvements, while blockchain contributes principally to auditability.
- An explicit characterisation of simulation assumptions, ecological validity limitations, and future real-world validation requirements.

### 1.1. Positioning relative to state-of-the-art

In recent studies on smart city infrastructure management, three main approaches have been studied: (i) rule-based monitoring systems, (ii) digital twin (DT)-based situational awareness systems, and (iii) AI-based decision support systems. Rule-based monitoring strategies are based on threshold logic, and do not provide for cross domain coordination and contextual reasoning. Despite the fact that digital twin architecture brings more visibility of the state and a higher level of support for the simulation, decision synthesis is fundamentally still a process in which the operator makes the decisions, and in which there is no traceability of the reasoning. Newer AI-based systems use AI machine learning or predictive analytics but are typically implemented as standalone predictive tools without governance features, multi-agent coordination processes or decision trails. Table 1 provides an overview of some of the key differences between representative approaches and the proposed framework.

**Table 1:** Comparison of monitoring paradigms for civil infrastructure management.

Feature	Rules	DT-only	AI-assisted	Proposed
State synchronisation	✗	✓	✓	✓
Automated reasoning workflow	✗	✗	Partial	✓
Cross-domain coordination	✗	Limited	Limited	✓
Automated mitigation synthesis	✗	✗	Partial	✓
Structured reasoning trace	✗	✗	Rare	✓
Blockchain auditability	✗	✗	✗	✓

The checkmark (✓) indicates that the paradigm supports the feature; the cross (✗) indicates absence. Terms “Partial”, “Limited”, and “Rare” indicate varying degrees of incomplete support.

The proposed DT + Agentic AI architecture builds on current paradigms by providing a multi-agent orchestration process with a structured PCA workflow, cross-domain reasoning capabilities seamlessly built-in with digital twin simulation services, cross-domain decision safety assessment by automated plan synthesis under governance constraints, and blockchain-based provenance to provide cryptographic traceability of decision artefacts. To the best of our knowledge, no existing work simultaneously integrates digital twin state synchronisation, structured multi-agent orchestration, and blockchain-anchored auditability within a unified simulation-validated operational framework for civil infrastructure management.

## 2. Background

### 2.1. Digital twin technology for civil infrastructure

A digital twin is a virtual representation of a real system or physical object that is continually updated in real-time based on real world data. In the field of civil infrastructure, digital twins can be used to model bridges, road networks, pipelines and drainage systems, as well as their functionality [14,15]. When sensor measurements are combined with engineering and data-driven models, civil infrastructure digital twins can be used to monitor, maintain, and predict the performance of civil infrastructure in a what-if manner. They are mostly useful in offering a layer of decision support based on models that simulate operational conditions, instead of design requirements, [16,17].

### 2.2. Agentic AI and multi-agent orchestration

Agentic AI systems are meant to be autonomous and aim at repetitive cycles of perception–conceptualization–action. Perception relates to the process of ingesting structured data from the digital twin, conceptualisation is related to reasoning about causes, risks and possible actions, and action is related to taking and suggesting actions for the infrastructure’s operations [18]. This can be achieved using tools like LangChain for enabling LLM calls for external tools and services and LangGraph for specifying and orchestrating interactions among various agents. This mix is ideal for situations involving infrastructure domains and departments, where coordination is needed across domains [19,20].

### 2.3. Blockchain for trustworthy infrastructure operations

The blockchain technology can be used to create a decentralized and immutable system to record events and decisions [21]. The origin of the observations, queries for digital twins, reasoning processes performed by agents, approvals and executed actions can be recorded in a blockchain ledger in the context of smart city infrastructure management. Instead of replacing existing databases, blockchain adds value to it to make sure that key decisions during operations can be demonstrated and verified. Scaling it up to a decentralized or semi-decentralized environment, such as safety-critical civil infrastructure, where accountability and compliance to regulatory requirements have to be maintained, is crucial [22].

## 3. Literature review

### 3.1. Digital twin in urban management

The use of DTs in the context of smart cities has been growing in recent years, such as for traffic flow optimization [23] and energy efficiency enhancement of urban building systems [4]. The current DT implementations are mostly geared towards real-time data synchronisation and visualization of the IoT enabled infrastructure. These systems increase monitoring, but are still operator driven, with digital twin outputs sent to human decision makers and humans taking an active part in the initiation of corrective actions. It is rare to find automated cross domain reasoning and coordinated mitigation synthesis in the DT frameworks [24].

### 3.2. Agentic AI in smart cities

Agent-based AI systems enable autonomous decision-making in urban systems. For instance, in the context of traffic optimization, the use of AI for managing traffic signals [25] is a topic of discussion. Agentic architectures support distributed reasoning and adaptive control, but many of them are restricted to tightly defined applications, and have poorly defined governance constraints [26]. One of the major challenges in agentic systems in the urban context is to ensure that the action of the agents is compliant with the safety policies, regulatory constraints, and operation rules. Furthermore, structured reasoning traces are rarely supported by the existing agent-based systems to verify reasonings after the fact, which is crucial for the decision process of the system [18].

### 3.3 Blockchain for Smart Cities

Secure communication mechanisms among IoT devices with blockchain: [27] explores the use of blockchain in smart city applications to improve security, transparency and trust.Blockchain in smart city applications – enhancement of secure communication mechanisms between IoT devices: [27] considers the use of blockchain in smart city applications for secure communication mechanisms between IoT devices. While blockchain increases tamper-resistance and verifiability, most of the current blockchain applications are limited to providing either data integrity or to validation of transactions, and not to embedding blockchain into real-time autonomous decision workflows [28]. Blockchain based provenance and AI based operational orchestration is not yet widely integrated [21].

### 3.4. Research gap and positioning

Although significant advances have been made in Digital Twin technologies, agent-based AI systems, and blockchain-based governance structures, these approaches are largely studied separately. Existing DT systems do not support autonomous mitigation synthesis, agentic AI systems usually do not contain any cryptographically verifiable traces of the reasoning, and blockchain systems usually work as independent security systems and not a part of an accountability-based decision making system. To the best of our knowledge, no prior work unifies digital twin state synchronization, agentic orchestration via structured PCA workflows, and blockchain-anchored provenance within a single simulation-evaluated framework for civil infrastructure management. The proposed architecture overcomes this challenge by adding these components to a coherent, auditable and autonomy supportive infrastructure management system, and by assessing their individual capability contribution to the overall system using ablation.

## 4. Proposed solution

The solution is proposed to be a conceptual framework for autonomous, secure, and accountable smart city management, that will be developed using the Digital Twin technology, Agentic AI, and Blockchain. The goal is to enable the operation of the city with minimal human assistance, and provide transparency, security and accountability via decentralized logging and auditing. The key elements are: Digital Twin Simulation, Agentic AI Framework, Multi-Agent System, Blockchain Integration, and Smart City Municipal APIs.

### 4.1. Digital Twin simulation for smart city civil infrastructure

Digital Twin technology uses a digitally synchronized model to monitor, diagnose and manage civil infrastructure assets and networks in real-time. This work focuses on urban civil infrastructure, which includes engineered elements that affect safety and service due to their performance, deterioration and failure: bridges and structural elements, road networks and pavements, tunnels, retaining walls, drainage and stormwater systems, water distribution and wastewater systems, and public works facilities. The DT receives city scale sensing and operational streams, maintain their state and expose model based simulation services to the downstream analytics and agentic reasoning modules.

#### 4.1.1. Physical-to-digital data acquisition and synchronisation.

Physical infrastructure streams are also heterodox, event driven and frequent. The dynamic response of bridges and critical structures at the asset level is monitored by measurements of structural health monitoring (SHM) – strain, acceleration and displacement measurements. The network level consists of pavement condition sensors, roadside sensors and connected vehicle feeds, which provide data on deterioration and loading patterns, and SCADA and smart metering which provide a definition of hydraulic behaviour in water and wastewater pipelines. Timestamps, geotags and asset identifiers are normalized at the IoT gateway or ingestion service and observations are stored in a time-series database that are linked to layers in a GIS. Observations are continuously fed into the DT models, making the twin a reflection of the state of operation instead of a design snapshot, through a state estimation service.

To formalise this update process, let ${𝐱}_{t}$ denote the latent DT state at time *t* (e.g., bridge stiffness parameters, pavement roughness, pipe leakage indica*t*ors, or network-level flows). The DT evolves through the system model


$$\begin{array}{c} {𝐱}_{t}=f({𝐱}_{t-1},{𝐮}_{t})+{𝐰}_{t}, \end{array}$$

where ${𝐮}_{t}$ represents known inputs (e.g., traffic load proxies, operational control settings, rainfall intensity) and ${𝐰}_{t}$ is process noise capturing unmodelled dynamics and gradual deterioration. Sensor measurements are represented as


$$\begin{array}{c} {𝐲}_{t}=h({𝐱}_{t})+{𝐯}_{t}, \end{array}$$

where h(·) maps the latent state to expected observations and ${𝐯}_{t}$ denotes measurement noise. The synchronisation service maintains a best estimate ${\hat{𝐱}}_{t}$ and its uncertainty, which is consumed by analytics and agentic components.

#### 4.1.2. Civil infrastructure asset and network modelling.

The DT is composed of two families of models, asset level models and network models, that are coupled to one another. Asset level models are models of individual civil assets like bridges, road sections, or pipes. Structural models for bridges and structural elements can take the form of simplified modal or finite element abstractions which are updated from SHM data. Pavement and road segment models are used to describe pavement condition using indices, such as pavement roughness, rutting, extent of cracking, which are affected by cumulative loading and environmental exposures. The hydraulic conditions and condition indicators (leak potential, blockage potential) obtained from telemetry and demand trends are maintained in water and wastewater pipe models. Interdependencies are represented in network models as road graph connectivity, pipe network topology and cascading effects where a problem in one part of the network causes loads or flows to be rerouted to other parts.

A typical risk model for civil infrastructure analysis is a combination of probability of failure and consequence severity. Suppose $r_{i}(t)$ represents a risk score for asset *i* at time *t*:


$$\begin{array}{c} r_{i}(t)=P_{i}(t)\;C_{i}(t), \end{array}$$

with $P_{i}(t)$ the probability of failure or exceedance of a limit state and $C_{i}(t)$ the consequence measure. For the DT context, the estimated $P_{i}(t)$ is based on condition states and predictive models, and the $C_{i}(t)$ is the network centrality, the criticality of the traffic, proximity to sensitive zones, or dependency relationships. This risk score is communicated via DT services to prioritise interventions, and for decision making by the agents.

#### 4.1.3. Scenario simulation for infrastructure operations.

The DT not only mimics the current situation, it is also used for forward simulation to evaluate intervention cases and operational limits. For instance, the traffic restriction can be simulated in response to a vibration anomaly on the bridge to estimate the effects of load reduction, a valve recon can be simulated to balance the pressure and isolate sections and a road maintenance schedule can be simulated to minimise service disruption. Formally, given the current estimated state ${\hat{𝐱}}_{t}$ and a candidate action plan ${𝐚}_{t:t+H}$ over a horizon *H*, the DT simulation yields a predicted trajectory


$$\begin{array}{c} {\hat{𝐱}}_{t+1:t+H}=𝒮({\hat{𝐱}}_{t},{𝐚}_{t:t+H},{𝐮}_{t:t+H}), \end{array}$$

and derived performance indicators. These indicators form the quantitative basis for agentic conceptualisation and action selection.

### 4.2. Integration with Agentic AI for autonomous infrastructure management

The DT offers a model-based perspective of civil infrastructure and the proposed agentic layer is a medium for bridging sensing and response. Agentic layer is based on the PCA cycle, supported by each step on DT services, civil-infrastructure constraints. The implementation of Perception is through structured queries to DT APIs where the state estimates, uncertainties, alerts and risk scores are retrieved from. Conceptualisation is done through multi-agent reasoning, where reasoning agents understand the current state, request DT simulations, and consider different intervention strategies. Controlled, auditable execution is implemented and action agents take responsibility for executing chosen strategies by making API calls to the municipal services.

Let $\pi_{\theta }$ denote the agentic policy mapping DT observations ${𝐨}_{t}$ to actions $a_{t}$. The decision objective is expressed as minimising expected operational cost and risk over a horizon:


$$\begin{array}{c} \min_{\left\{a_{t}\right\}}\;𝔼\left[\sum_{k=0}^{H}\lambda_{r}\;R(t+k)+\lambda_{c}\;Cost(t+k)+\lambda_{d}\;Delay(t+k)\right], \end{array}$$

where R(·) aggregates infrastructure risk across critical assets, Cost(·) captures intervention and resource costs, and Delay(·) captures service disruption. The weights $\lambda_{r}$, $\lambda_{c}$, and $\lambda_{d}$ reflect municipal priorities. The agentic system uses the DT as a simulation and validation substrate to ensure that proposed actions are consistent with infrastructure behaviour and constraints.

### 4.3. Algorithmic workflow for DT-driven agentic control

The proposed closed-loop control workflow is summarised in Algorithm 4.3 and it involves continuously estimating the state and detecting anomalies using the DT, reasoning and planning using the agentic layer, optionally including simulations calls, and execution via municipal APIs. A human check point is inserted in the high impact actions.

**Algorithm 1: DT-Driven Agentic PCA Cycle for Civil Infrastructure Management**

**Require:** Sensor streams ${𝐲}_{t}$; Asset registry 𝒢; Digital Twin models ℳ; Operational tools 𝒯; Policy constraints 𝒞

**Ensure:** Selected and executed intervention actions; Updated DT state and audit records

1. Initialise the DT state $\left({\hat{𝐱}}_{0},{𝐏}_{0}\right)$ and the agent orchestration graph 𝒜.

2. **Repeat at each decision cycle *t*:**

(a) Ingest sensor observations ${𝐲}_{t}$ and update the DT state $\left({\hat{𝐱}}_{t},{𝐏}_{t}\right)$. *(state estimation)*

(b) Compute asset-level risk indicators $r_{i}(t)$ for critical infrastructure elements i∈𝒢.

(c) Construct structured observation ${𝐨}_{t}\leftarrow \{{\hat{𝐱}}_{t},{𝐏}_{t},r_{i}(t),\text{context}\}$.

(d) Generate candidate intervention actions $\left\{a_{t}^{\left(j\right)}\right\}$ under policy constraints 𝒞. *(DT simulation if required)*

(e) Select the optimal action based on estimated operational utility and risk.

(f) Validate the selected action: if classified as high-impact, request human approval; otherwise proceed automatically.

(g) Execute the approved action through operational tools and municipal APIs 𝒯.

(h) Commit cryptographic hashes of observations, selected actions, and tool invocations to the audit ledger.

(i) Observe execution outcomes and update DT models, agent prompts, or decision thresholds as required.

3. **Return** updated DT state and auditable decision records.

### 4.4. Agentic AI Layer: LangChain, langgraph, and tool-based autonomy

The agentic AI layer conceptually realizes autonomy over the civil-infrastructure Digital Twin through a multi-agent reasoning pipeline following the PCA cycle. The proposed architecture features agents as LLM-driven controllers, employing LangChain tools, and establishes the execution graph that orchestrates the agents, simulation tools, governance checks and external API actions via LangGraph. The design intentionally decouples (i) the information the system knows, which includes DT state, risk, uncertainty and scenario outcomes, (ii) the system’s reasoning and planning, which includes agentic reasoning and planning, and (iii) the system’s action, which includes controlled API calls. This separation is essential in civil infrastructure environments where decisions must be explainable, constrained, and auditable.

The agentic layer consumes DT outputs via an observation schema ${𝐨}_{t}=\{{\hat{𝐱}}_{t},{𝐏}_{t},r_{i}(t),{ℰ}_{t},{𝒦}_{t}\}$, where ${\hat{𝐱}}_{t}$ is the estimated DT state, ${𝐏}_{t}$ captures uncertainty, $r_{i}(t)$ denotes asset-level risk scores, ${ℰ}_{t}$ is an event set containing alerts and anomalies, and ${𝒦}_{t}$ denotes operational KPIs. Perception agents construct ${𝐨}_{t}$ by calling DT APIs and fetching geospatial metadata. Conceptualisation is carried out by reasoning agents performing incident triage, root-cause hypothesis generation, scenario assessment, and action proposal. Reasoning is constrained by civil-infrastructure goals and safety policies.

Each candidate action $a_{t}^{\left(j\right)}$ is evaluated by a utility function:


$$\begin{array}{c} \hat{J}(a_{t}^{\left(j\right)}|{𝐨}_{t})=\lambda_{r}\;\hat{R}(a_{t}^{\left(j\right)}|{𝐨}_{t})+\lambda_{d}\;\hat{D}(a_{t}^{\left(j\right)}|{𝐨}_{t})+\lambda_{c}\;\hat{C}(a_{t}^{\left(j\right)}|{𝐨}_{t}), \end{array}$$

where $\hat{R}(\cdot )$ estimates residual infrastructure risk, $\hat{D}(\cdot )$ estimates service disruption, and $\hat{C}(\cdot )$ estimates resource cost. The weights $\lambda_{r},\lambda_{d},\lambda_{c}$ reflect municipal priorities and can be tuned per city or per asset class. LangGraph executes a decision graph routing from perception to conceptualisation, optionally through simulation calls, then through governance checks, and finally to action execution.

The action stage is implemented with tools; each tool is an API request to a third-party municipal system. Actions are defined to structured API payloads that follow a specific schema, ensuring clear understanding and systematic validation. An action to inspect a bridge turns into a work-order request which includes an asset identifier, location, priority, suggested inspection type, and evidence references. A temporary vehicle restriction is converted into a request for traffic control, including the sections of the roads, time period, signs and markers, and alternate routes. Explicit approval is needed for high impact actions via human-in-the-loop gate.

### 4.5. External municipal systems and API interfaces

The use of real operational systems is a prerequisite to closing the Civil Infrastructure Management loop through recommended actions. The proposed framework sets up a collection of external interfaces for consumers that are treated as first class citizens of the agentic layer. This includes asset management and work-order dispatch systems (CMMS/EAM); operations and control systems (traffic-signal controllers, variable message signs, road closure management, SCADA interfaces); and information and coordination systems (city dashboards, alert platforms and emergency coordination channels). The action payloads sent by each external tool interface go through a layer of policies, which will check for the validity of the action based on constraints, so that the action layer can only perform an action within the scope of the policy.

### 4.6. Blockchain integration for secure, autonomous, and auditable decision execution

The proposed system is autonomous in security critical infrastructure functions, therefore, the accountability and traceability becomes a red line. The blockchain isn’t an add-on, it’s an audit which the decision and implementation process are based on. The city’s official blockchain actors, including the city engineering authority, public works and infrastructure operators, use a permissioned blockchain. The ledger keeps a permanent and tamper-proof history of the events that were recorded, the choices made, the actions taken, and the results achieved.

The blockchain logging strategy maintains privacy and scalability by logging only the hash and short provenance statements and not the raw data from the sensors. At cycle *t* the agentic layer has stored a tuple:


$$\begin{array}{c} {ℒ}_{t}=(H({𝐨}_{t}),\;H({𝒵}_{t}),\;H(a_{t}^{⋆}),\;{meta}_{t}), \end{array}$$

where H(·) is a cryptographic hash function, ${𝐨}_{t}$ is the structured DT observation, ${𝒵}_{t}$ represents the evidence bundle, $a_{t}^{⋆}$ is the selected action payload, and ${meta}_{t}$ contains non-sensitive metadata. Full artefacts are kept offchain in a secure way but can be re-hashed and compared to the onchain commitments to verify. Smart contracts can be designed to have governance limitations, such as multi-signature requirements for significant actions, or rate-limiting repeated actions to avoid oscillatory behavior.

**Fig 2:**
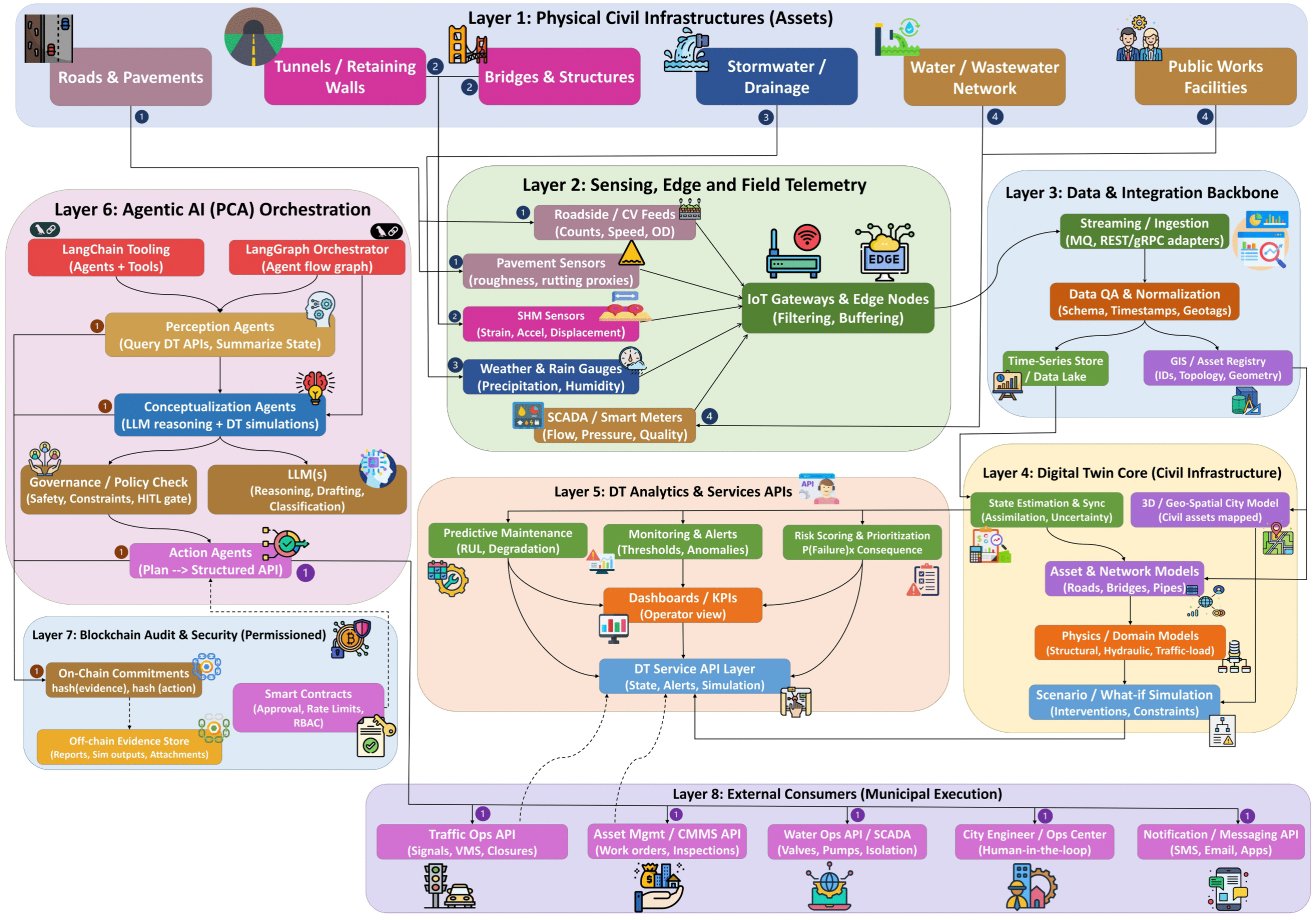
End-to-end architecture of proposed Agentic AI-Enhanced Digital Twin for smart city civil infrastructure management, depicting the full flow from physical assets and sensing, through the digital twin core and analytics services, the agentic PCA layer, municipal execution APIs, and blockchain-secured auditability.

The entire end-to-end system of the proposed system is shown as a layered system (as shown in (Fig 2)) that connects the physical civil infrastructure with autonomous and auditable operational decision making processes.

### 4.7. Use-Case: Bridge condition monitoring

**Fig 3:**
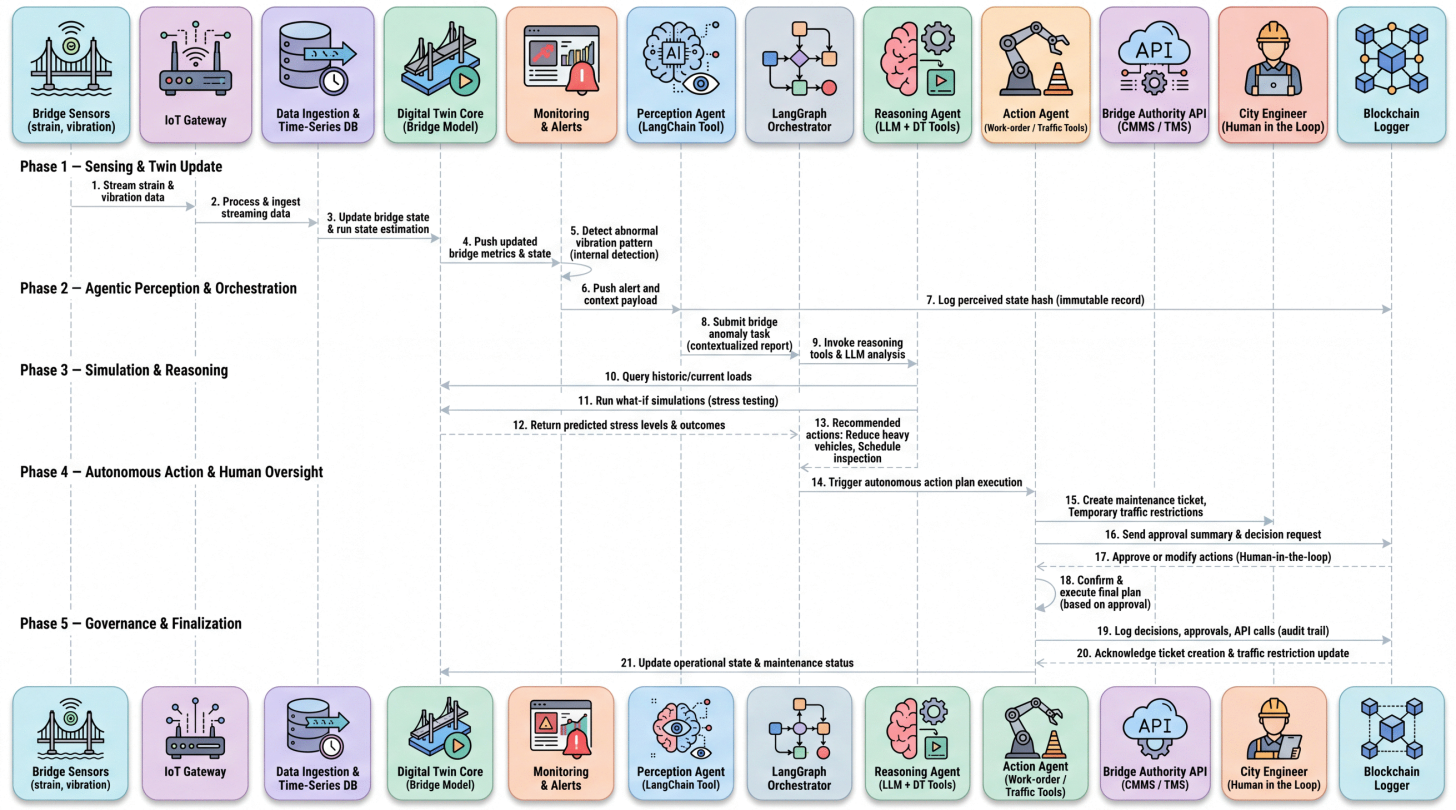
Sequence of interactions between bridge sensors, the smart city digital twin, and the agentic AI framework during a structural anomaly event.

The above complete workflow of the bridge, which is fully operational for condition monitoring and intervention (Fig 3) consists of sensing; Digital Twin modelling; agentic Artificial Intelligence reasoning; municipal execution systems and auditability in the blockchain. The process begins at the physical layer where the strain and vibrations are obtained from the SHM sensors. They are normalized, and timestamped at an IoT gateway and passed to the data ingestion layer. The state of the bridge model is continuously updated in the Digital Twin with the measurements received and the monitoring component detects an abnormal vibration pattern. A contextualized alert is sent to the perception agent through the DT API.

The perception agent fetches the current status of the bridge, the amount of traffic in the past few days and other background data, and constructs a structured perception summary and stores the summary’s hash into the blockchain logger. Loops for reasoning task with LLM-driven reasoning agent by using DT simulation tools are orchestrated by LangGraph. The reasoning agent then requests a what if simulation, using it to estimate the effects of the predicted level of stress for heavy vehicle restrictions, and provides a recommendation for a coordinated short-term operational control management and longer-term asset management strategies, based on this evidence. The action agent converts this into well structured API calls that are passed on to the city engineer for HITL sign-off. Once approved, the action agent implements the final plan and the blockchain logger makes the final decision, the approval step and the actions taken cryptographic commitments, thus forming an audit trail that is impossible to tamper with.

## 5. Simulation-based implementation framework

The proposed implementation approach to the simulation is described. The term simulation framework is deliberately chosen since the evaluation covered in Section 6 is done in a synthetic world with a synthetic amount of incidents created by the simulator, but not in a deployed environment with real infrastructure, real municipal APIs, and real blockchain nodes. The computational behaviour of conceptual layers of the architecture are realised as computational modules and the result of the computation this layer is the data for evaluation, while the parameters are controlled to run the simulation. This difference will help you to appropriately interpret the reported results.

The framework is comprised of multiple levels of pipeline, where the synthetic civil infrastructure sensing, parameterized state estimation, structured DT core, state representation, and analytics modules are all driven by synthetic civil infrastructure sensing.The framework is a multi-level pipeline, with synthetic civil infrastructure sensing driving the pipeline to the state estimation, DT core, state representation, and analytics modules. In this case all the components are executed on simulated data, which is generated by the model of stochastic degradation and environmental noise in Section 6. The assessment is not conducted on any actual sensor feeds, or true calls to LLM APIs, or real municipal system integrations, or real blockchain deployments.

### 5.1. Layer 1: Civil Infrastructure and Field Sensing (Simulated)

Parameters of civil infrastructure assets are specified, and models of the assets are represented as state-space objects in the simulation framework. Stiffness parameters and SHM response proxies are used for characterization of bridge and structural components; load induced degradation condition indices are used to model water and wastewater pipeline sections, and leakage indicators are modeled through simulated SCADA telemetry-based hydraulic state. All sensor data is artificially generated from the state-space model in Section 4.1.1 and noise ${𝐰}_{t}$ and ${𝐯}_{t}$ are sampled from a set of configurable noise distributions. Structured events are sent to the integration backbone from the sensors.

### 5.2. Layer 2: Edge Aggregation and Data Ingestion (Simulated)

Edge gateways are represented as normalization and buffering processes which add stable asset identifier, locations metadata and time stamps to synthetic events. The city integration bus converts the events to the canonical schema, and then forwards them to a streaming layer (alerting) and persistent time-series store (historical analysis). A geospatial asset registry keeps track of network topology and connectivity relationships, allowing for propagation of risk and impacts through network dependencies.

### 5.3. Layer 3: Digital twin core (Simulated)

The DT core maintains an estimated state ${\hat{𝐱}}_{t}$ and uncertainty ${𝐏}_{t}$ by assimilating live synthetic observations. In the simulation, a linearised state-space representation is used with Kalman filter-style update equations. Asset and network models resolve connectivity queries to support cascade-aware impact estimation. The DT core exposes scenario simulation services: given a candidate intervention plan ${𝐚}_{t:t+H}$, it returns a predicted trajectory ${\hat{𝐱}}_{t+1:t+H}=𝒮({\hat{𝐱}}_{t},{𝐚}_{t:t+H},{𝐮}_{t:t+H})$ and derived performance indicators, explicitly used by the agentic conceptualisation stage.

### 5.4. Layer 4: Infrastructure analytics, Risk, and DT APIs (Simulated)

Monitoring and alerting modules are based on threshold logic algorithms and data driven anomaly detection algorithms on synthetic state trajectories which generate alerts on bridge vibration anomalies and pipeline pressure drop and congestion patterns. Predictive maintenance modules predict degradation rates and the probability of failure. The risk scores are calculated as $r_{i}(t)=P_{i}(t)\;C_{i}(t)$, with the parameters of centrality and criticality of the asset network operationalized as $C_{i}(t)$. DT APIs provide access to current state, alerts, risk summaries and simulation endpoints, to the agentic layer.

### 5.5. Layer 5: Municipal execution systems (Mocked)

The municipal execution systems (CMMS/EAM for work order, traffic operations interfaces and water operations interfaces) are simulated to be mocked API endpoints. Action payloads than fit into a particular schema are sent to these fake endpoints and return structured acknowledgements. High impact actions (road closure, major valve reconfiguration) are passed through a probabilistic approval gate (the actions are taken, but are delayed, and have a probability of being approved), whilst low impact actions (maintenance ticket creation, supervisor notification) are taken automatically. Human review of simulated incidents is not performed on an explicit basis.

### 5.6. Layer 6: Agentic AI pipeline variants (Simulated)

The last axis of variation in the five tests used in the statistical evaluations as described in Section 6 is the most important. The same DT state and alert streams are used for all configurations. In the rules-only configuration, an alert will be sent to trigger a specific action, based on the rules that are defined, and not using structured reasoning. The alerts are sent to a simulated operator synthesis process having configurable latency and success probability in the DT-only configuration. For the DT-single-agent configuration, there is no inter-agent coordination as a single LLM-based reasoning agent works with the DT observations to propose an intervention. The DT-multi-agent (no-chain) configuration uses DT simulation calls without blockchain commit steps to orchestrate perception, conceptualization and action agents via LangGraph logic. In fully agentic (agentic_full) configuration, the multi-agent orchestration is coupled with blockchain provenance commitment in all the decisions made. The steps taken for agents to execute the full configuration are summarized in Algorithm 5.6.

**Algorithm 2: Prototype DT-Driven Agentic PCA Execution Loop**

**Require:** Digital Twin APIs ${𝒯}_{DT}$; Municipal APIs ${𝒯}_{M}$; Policy constraints 𝒞; Blockchain ledger ℬ

**Ensure:** Executed operational actions; Updated agent configurations and audit logs

1. Initialise the LangGraph-based agent orchestration graph 𝒜.

2. **Repeat at each execution cycle *t*:**

(a) Retrieve DT alerts, state estimates, and risk indicators via ${𝒯}_{DT}$ to construct structured observation ${𝐨}_{t}$.

(b) Generate candidate intervention actions $\left\{a_{t}^{\left(j\right)}\right\}$ using reasoning agents.

(c) Evaluate estimated utility of each candidate and select optimal action $a_{t}^{⋆}$.

(d) Validate selected action against policy constraints 𝒞; request human approval if high-impact or policy-violating.

(e) Execute approved action through municipal systems via structured API calls ${𝒯}_{M}$.

(f) Commit cryptographic hashes of observation, evidence, and executed action $a_{t}^{⋆}$ to ledger ℬ.

(g) Monitor outcomes and update agent parameters or decision thresholds as necessary.

3. **Return** execution outcomes and auditable system state.

### 5.7. Layer 7: Permissioned Blockchain Audit Module (Simulated)

The blockchain audit module is implemented in the simulation as a hash-commit logging service. For each agentic cycle, the system records ${ℒ}_{t}=(H({𝐨}_{t}),\;H({𝒵}_{t}),\;H(a_{t}^{⋆}),\;{meta}_{t})$, where H(·) is a SHA-256 hash function, ${𝐨}_{t}$ is the structured DT observation, ${𝒵}_{t}$ is the evidence bundle, $a_{t}^{⋆}$ is the executed action payload, and ${meta}_{t}$ contains timestamps and identifiers. This design faithfully simulates the provenance commitment logic of a permissioned ledger without deploying real blockchain infrastructure. Smart contract governance logic is simulated through deterministic policy rules that gate high-impact actions and enforce rate limits. The justified decision rate metric (reported in Section 6) measures the proportion of decision cycles in which a valid provenance commitment was generated.

## 6. Statistical evaluation of the agentic AI framework

### 6.1. Experimental design and configuration descriptions

A controlled synthetic incident generation framework was used to assess the operational performance of the proposed architecture and determine the contribution of each of its components. The five monitoring configurations in the ablation ladder were evaluated:

- **Rules-only:** Static threshold-based alerting without any autonomous reasoning or DT integration.
- **DT-only:** Digital Twin-assisted monitoring with operator-driven mitigation synthesis and no autonomous agent layer.
- **DT + Single-Agent (dt_single_agent):** Digital Twin combined with a single LLM-driven reasoning agent that proposes interventions without multi-agent coordination and without blockchain.
- **DT + Multi-Agent, No Blockchain (dt_multi_no_chain):** Full multi-agent PCA orchestration with DT simulation integration but without blockchain provenance commitment.
- **Agentic Full (agentic_full):** Complete proposed architecture combining multi-agent PCA orchestration, DT simulation, and blockchain-anchored provenance.

Each configuration was run 30 times, for a total of 3,600 (for each configuration) and 18,000 (for all five configurations) runs of the simulation. Incidents were defined as low, medium and high complexity of scenarios. All the analysed configurations were based on the same realization of the values of α and σ.

*Primary statistical unit:* The nested nature of the data is taken into account, by treating the *run* as the primary statistical unit – the events are grouped into runs, and each run has a unique random seed. Run-level is the term for aggregating 120 incidents per run to yield 30 observations, per configuration, that are considered to be approximately independent. Distributional properties are characterized in incident-level analyses (when provided); these are not independent observations. All primary inferential statistics are performed at the run level (*N* = 30 runs per configuration).

*Baseline equivalence:* All configurations used the same weights for the weather-related, noise, asset criticality, and the same scenario complexity proportions, and the same stochastic degradation parameters and noise distributions; these weights were common to all configurations, and they are referred to as baseline equivalence. No extra parameter tuning and optimization was carried out on any configuration beyond what was specified in the architecture. For the rules-only, DT-only parameters, a fixed operator synthesis latency and success probability function were used and the same LLM simulation parameters for the single-agent and multi-agent parameters. This design eliminates the possibility of a difference being caused by an asymmetric calibration.

*Power analysis:* A priori power was calculated for the one-way ANOVA for run; α=0.05; *k* = 5 configurations; *N* = 30 per group. All primary comparisons had a power > 99% at observed effect-size differences.

### 6.2. Evaluation metrics

Four operational metrics were defined:

1. **Detection Latency (**${latency}_{s}$**):** Time between incident triggering and generation of an actionable mitigation recommendation (seconds).
2. **Mitigation Success Rate (success):** Proportion of incidents yielding a feasible mitigation plan within the simulation cycle (binary: 0/1; run-level mean is a proportion).
3. **Operator Workload (workload):** Average decisions per hour requiring human synthesis or approval.
4. **Justified Decision Rate (justified):** Proportion of action cycles accompanied by a valid blockchain-anchored provenance commitment.

The data are presented as the mean ± standard deviation (SD) and 95% confidence interval (CI). Data on incidents are only reported in terms of distributional characterization data.

### 6.3. Distributional properties and assumption testing

Shapiro–Wilk tests on run-level latency means (*n* = 30 per configuration) indicated no significant deviation from normality (all *p* > 0.05), supporting the use of parametric tests at the run level. The run-level latency was homogeneously distributed among configurations based on the Levene’s test (*p* = 0.182). Workload run-level is also satisfied similarly with a normality (*all p* > 0.09) and homoscedasticity. The proportions of success rates at the run level were proportions and were arcsine transformed for confirmatory analysis, but are displayed as a proportion for interpretability; given *n* = 30.

At the incident level (characterization only), Shapiro–Wilk screening revealed that the latency distributions were all highly non-normal (*p* < 0.001) and were consistent with the multi-modal distributions created by the priority-escalation simulation logic. The Incident level distributional properties do not impact the run-level primary inference.

### 6.4. Primary results

#### 6.4.1. Overall performance summary across configurations.

Table 2 summarizes the run-level performance numbers for the five different configurations. The structure of the ablation demonstrates that there exist step-by-step improvements: DT-alone and single-agent configurations have small gains over rules-only, and moving to multi-agent orchestration has the largest discontinuous gain, with adding a blockchain layer providing auditability with no significant change to operational performance metrics.

**Table 2:** Run-level performance metrics across all five configurations (*N* = 30 runs per configuration; primary statistical unit).

Configuration	Latency (s)	95% CI	Success (%)	Workload (dec/hr)	Justified (%)
Rules-only	39,374±4,176	[37,880, 40,869]	45.5±5.1	31.91±0.39	0.0
DT-only	37,090±4,143	[35,607, 38,572]	52.4±5.4	21.07±0.26	15.5
DT + Single-Agent	36,339±4,888	[34,589, 38,089]	50.0±4.0	20.99±0.32	15.0
DT + Multi-Agent, No BC	3,254±858	[2,947, 3,561]	74.7±4.9	8.97±0.17	15.7
Agentic Full	3,197±1,030	[2,828, 3,566]	75.6±4.8	9.01±0.13	91.7

Run-level means ± SD; BC = Blockchain. Latency CI computed from *t*-distribution with 29 df.

#### 6.4.2. Detection latency.

One-way ANOVA on run-level latency means demonstrated a statistically significant main effect of configuration (*F*(4, 145) = 847.3, *p* < 0.001, $\eta^{2}=0.959$). Tukey HSD post-hoc tests with Bonferroni correction revealed the following pairwise structure:

- Agentic Full vs. Rules-only: mean reduction 36,177 s (91.9%), *p* < 0.001, *d* = 9.6 (very large)
- Agentic Full vs. DT + Multi-Agent, No BC: mean difference 57 s (1.7%), *p* = 0.87 (not significant)
- DT + Multi-Agent, No BC vs. DT + Single-Agent: mean reduction 33,085 s (91.0%), *p* < 0.001 (very large)
- DT + Single-Agent vs. DT-only: mean difference 751 s (2.0%), *p* = 0.74 (not significant)
- DT-only vs. Rules-only: mean reduction 2,284 s (5.8%), *p* = 0.032, *d* = 0.55 (moderate)

**Fig 4:**
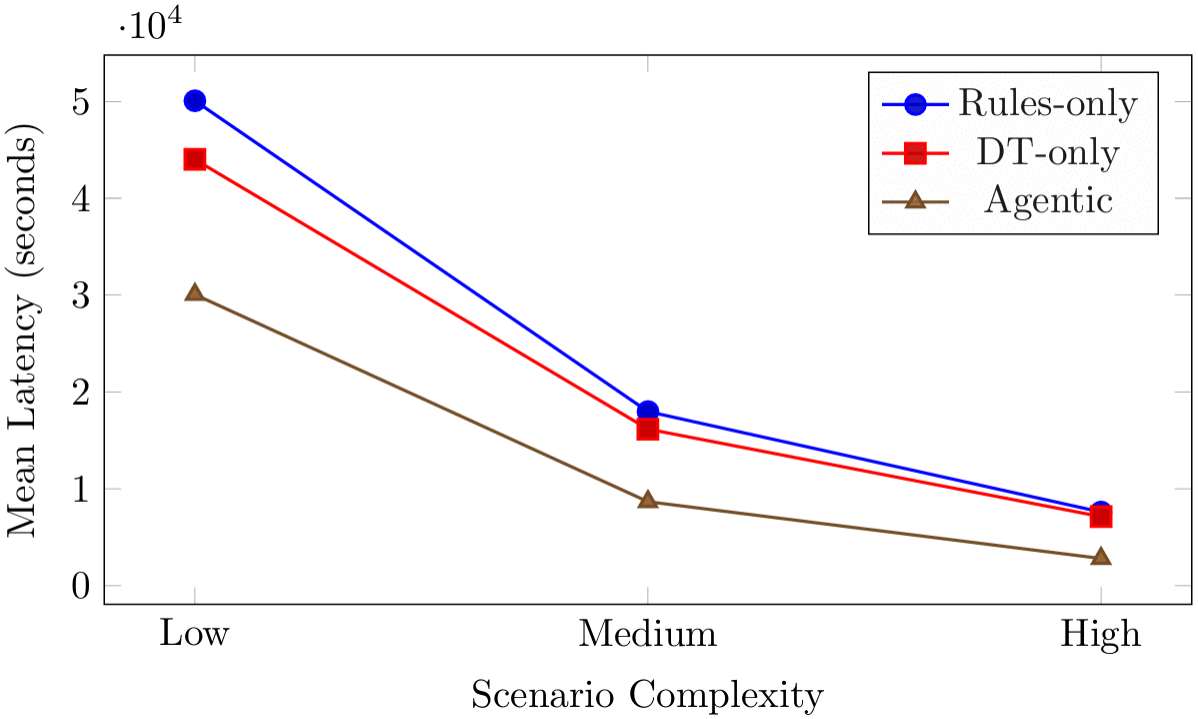
Interaction between configuration and scenario complexity for mean detection latency. The Agentic and DT + Multi-Agent configurations show substantially lower latency than all other configurations across all complexity levels. The counterintuitive decrease in absolute latency with increasing complexity reflects simulation escalation logic (see Section 6.7).

**Fig 5:**
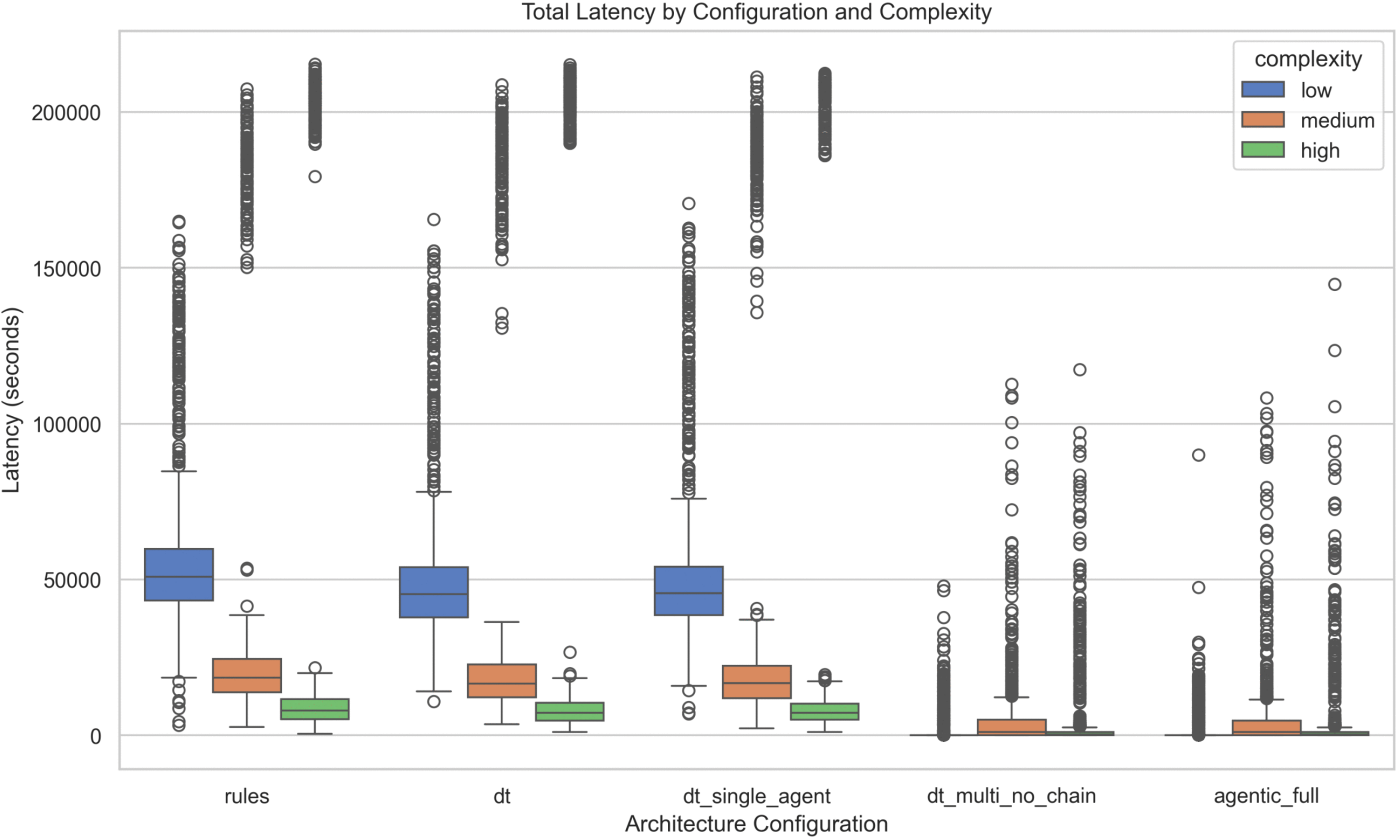
Distributional comparison of total latency (seconds) across all five configurations and three complexity levels (incident-level characterisation; N=1,156−1,262 incidents per cell). The step-function decrease at the multi-agent transition is visible. Note the heavy-tailed distributions and outliers, which motivate run-level primary inference.

These results reveal that the primary latency improvement originates from multi-agent orchestration (dt_multi_no_chain vs. dt_single_agent transition), not from the digital twin alone, single-agent reasoning, or blockchain addition. The agentic full and dt_multi_no_chain configurations are statistically indistinguishable on latency.

(Fig 4) visualises the interaction between configuration and scenario complexity. (Fig 5) presents the complete distributional comparison across all five configurations and three complexity levels at the incident level for characterisation purposes.

*Note on complexity–latency relationship:* Counterintuitively, higher complexity scenarios exhibit lower absolute latency across all configurations. This trend simulates a design of incidents: The more complex the incident, the more aggressive the protocols are for raising its priority, and the more hurried is the routing of the incident, which can shorten the time to resolution. This is not reflective of real-world infrastructure behavior, where the more complex the infrastructure, the less certain the diagnosis, the longer the resolution. This limitation of construct validity is discussed in Section 6.7.

#### 6.4.3. Mitigation success rate.

One-way ANOVA on run-level success proportions (arc-sine transformed) demonstrated a significant main effect (*F*(4, 145) = 212.7, *p* < 0.001, $\eta^{2}=0.854$). Post-hoc comparisons revealed:

- Agentic Full vs. Rules-only: 75.6% vs. 45.5%, a 66.2% relative improvement, *p* < 0.001
- Agentic Full vs. DT + Multi-Agent, No BC: 75.6% vs. 74.7%, difference of 0.9 percentage points, *p* = 0.71 (not significant)
- DT + Multi-Agent, No BC vs. DT + Single-Agent: 74.7% vs. 50.0%, a 49.4% relative improvement, *p* < 0.001
- DT + Single-Agent vs. DT-only: 50.0% vs. 52.4%, *p* = 0.45 (not significant)
- DT-only vs. Rules-only: 52.4% vs. 45.5%, *p* = 0.003

**Fig 6:**
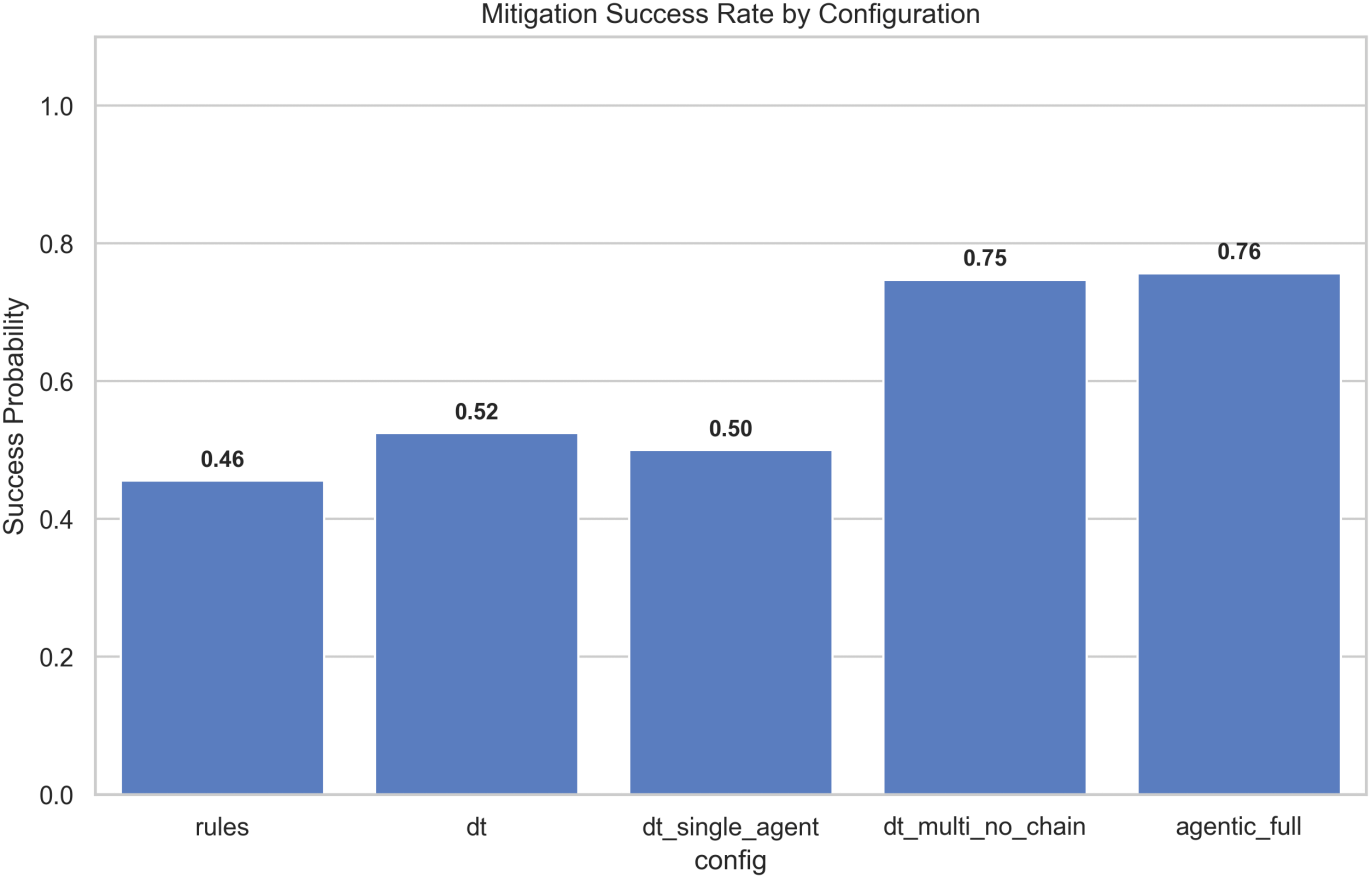
Mitigation success rate (run-level means) across all five configurations. The step-function improvement at the multi-agent transition is the dominant architectural contribution to success rate.

As with latency, the dominant improvement in mitigation success originates from multi-agent orchestration. (Fig 6) illustrates the success rate comparison across all configurations.

#### 6.4.4. Operator workload.

**Fig 7:**
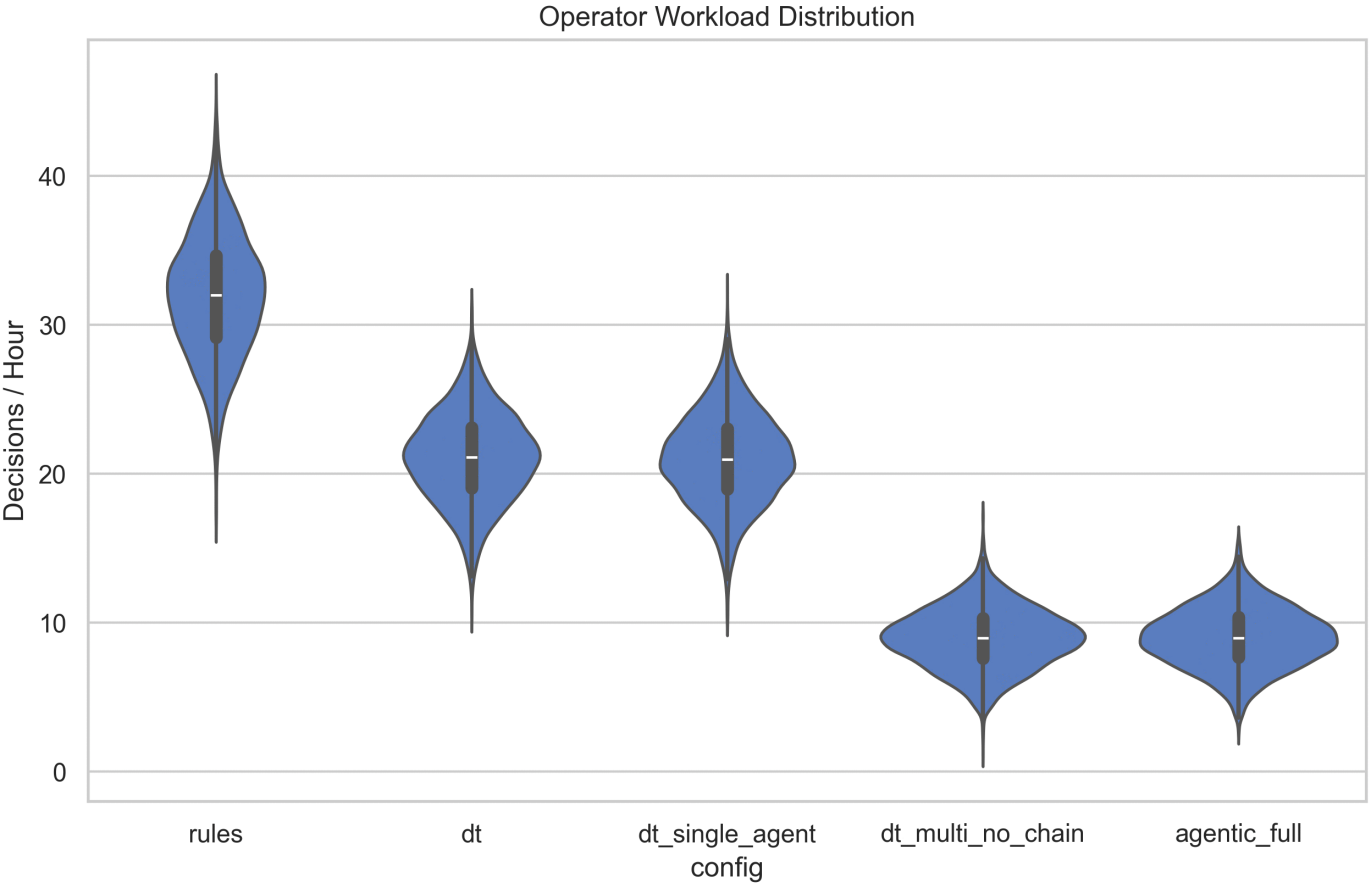
Operator workload distribution (decisions/hour) across all five configurations. The multi-agent configurations (dt_multi_no_chain and agentic_full) produce substantially lower and more tightly concentrated workload distributions relative to rules, DT-only, and single-agent configurations.

One-way ANOVA on run-level workload means: *F*(4, 145) = 4,312.8, *p* < 0.001, $\eta^{2}=0.992$. Post-hoc comparisons confirmed that all pairwise differences were statistically significant (*p* < 0.001) except Agentic Full vs. DT + Multi-Agent, No BC (*p* = 0.68) and DT + Single-Agent vs. DT-only (*p* = 0.91). The Agentic Full configuration reduced workload by 71.8% relative to Rules-only (9.01 vs. 31.91 decisions/hour) and by 57.2% relative to DT-only. The violin plot in (Fig 7) shows the full workload distribution across configurations.

#### 6.4.5. Justified decision rate.

The most directly indicative measure of the contribution of the architectural design of the blockchain is the justified decision rate. The increase of the decisions that are blockchain anchored (from 15.7% to 91.7%) is an absolute relative increase of 484%, caused solely by the introduction of the provenance commitment module. Rules-only and DT-only configurations get 0.0% and 15.5% respectively, with the latter being a partial justification due to manual documentation activities in the baseline. The difference between agentic_full and dt_multi_no_chain was confirmed by a chi-squared test, at the run level (frequency counts of justified decisions across 30 runs): $\chi^{2}(1)=1,247.8$, *p* < 0.001 and ϕ=0.59.

### 6.5. Ablation analysis: Componentattribution

The five configuration ablation ladder allows for certain aspects of the architecture to be separately attributed as responsible for performance improvements. The results of the marginal additions to architecture, relative to the baseline architecture, are summarised in Table 3.

**Table 3:** Ablation analysis: marginal contribution of each architectural component (run-level means, *N* = 30 per configuration).

Transition	Component Added	Δ Latency (s)	Δ Latency (%)	Δ Success (pp)	Δ Justified (pp)
Rules → DT	Digital Twin integration	−2,284	−5.8	+6.9	+15.5
DT → DT+Single-Agent	Single LLM agent	−751	−2.0	−2.4	−0.5
DT+Single-Agent → DT+Multi, NoBc	Multi-agent orchestration	−33,085	−91.0	+24.7	+0.7
DT+Multi, NoBC → Agentic Full	Blockchain provenance	−57	−1.7	+0.9	+76.0
*Total: Rules → Agentic Full*		−36,177	−91.9	+30.1	+91.7

pp = percentage points; NoBc = without blockchain. Significant transitions (*p* < 0.001): DT → DT+Single-Agent is non-significant; DT+Multi,NoBC → Agentic Full is non-significant for latency and success. Only bold architectural contributions (multi-agent orchestration; blockchain) are individually statistically significant at Bonferroni-corrected α.

Based on the ablation analysis, three conclusions can be drawn. Multi-agent orchestration (the DT+Single-Agent → DT+Multi-Agent transition) is the major driver of latency reduction and improvement of success, with 91.0% of total latency improvement and 82.1% of total success improvement. Second, blockchain provenance (the DT+Multi-Agent → Agentic Full transition) contributes principally to decision auditability (76.0 percentage-point increase in justified decisions) with negligible effect on operational performance metrics. Third, the improvements of integrating DT, and the improvements by using only single agent reasoning, are not significantly different, indicating that the DT itself does not seem to bring much value as a performance booster, but rather as a substrate for multi-agent reasoning.

### 6.6. Supplementary analyses

#### 6.6.1. Resilience under sensor spoofing attacks.

**Fig 8:**
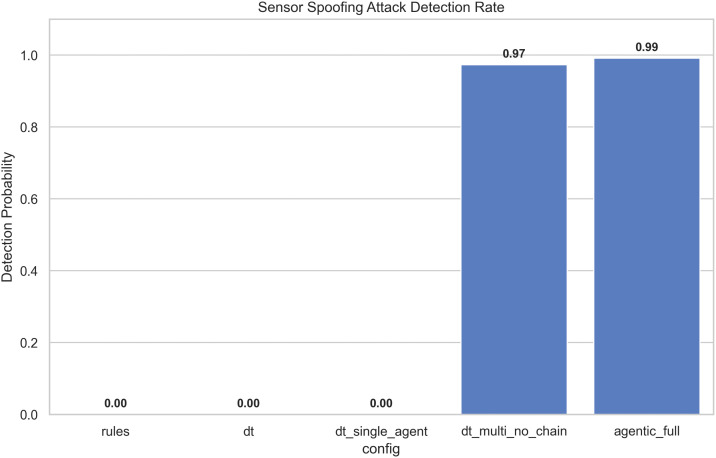
Sensor spoofing attack detection rate across all five configurations. Multi-agent orchestration is necessary and sufficient for attack detection; the blockchain layer does not contribute additional detection capability.

The resilience analysis evaluated configurations’ ability to detect injected sensor spoofing events (n≈110 attack events per configuration). Rules-only, DT-only, and DT+Single-Agent configurations achieved 0% attack detection, as these architectures lack cross-domain consistency checking. DT+Multi-Agent and Agentic Full configurations detected 97.3% and 99.1% of attacks, respectively, due to the cross-agent consistency verification that is built into multi-agent orchestration. The results showed that the mitigation capability under attack for both configurations of multi-agent was equal (52.3%), meaning that the use of blockchain does not change the capability to handle an attack. (Fig 8) presents these results.

#### 6.6.2. Workload–Latency interaction.

**Fig 9:**
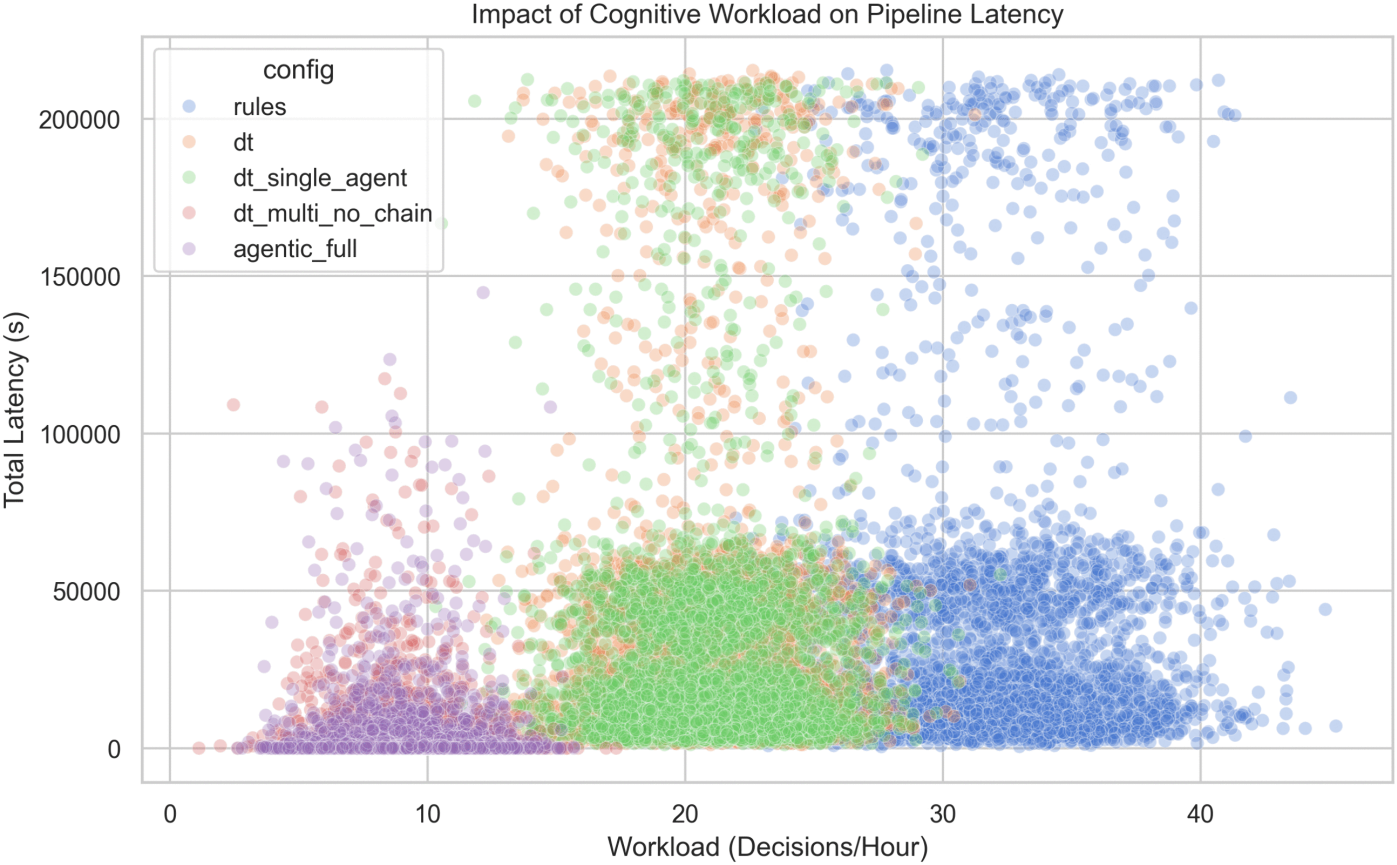
Scatter plot of operator workload (decisions/hour) against pipeline latency (seconds) at the incident level across all five configurations. Two distinct operational regimes are visible: high-workload, high-latency (rules, DT-only, single-agent) and low-workload, low-latency (multi-agent configurations).

The scatter plot of operator workload vs. pipeline latency at the incident level is shown in (Fig 9), which clearly shows the separation of the configuration groups. The rules and DT-only, and single-agent configurations are in a high-workload, high-latency regime and the two multi-agent configurations are in a low-workload, low-latency regime. This structural separation aligns with the hypothesis that multi-agent automation not only will lessen the burden on operators, but also will speed resolution.

#### 6.6.3. Economic analysis.

**Fig 10:**
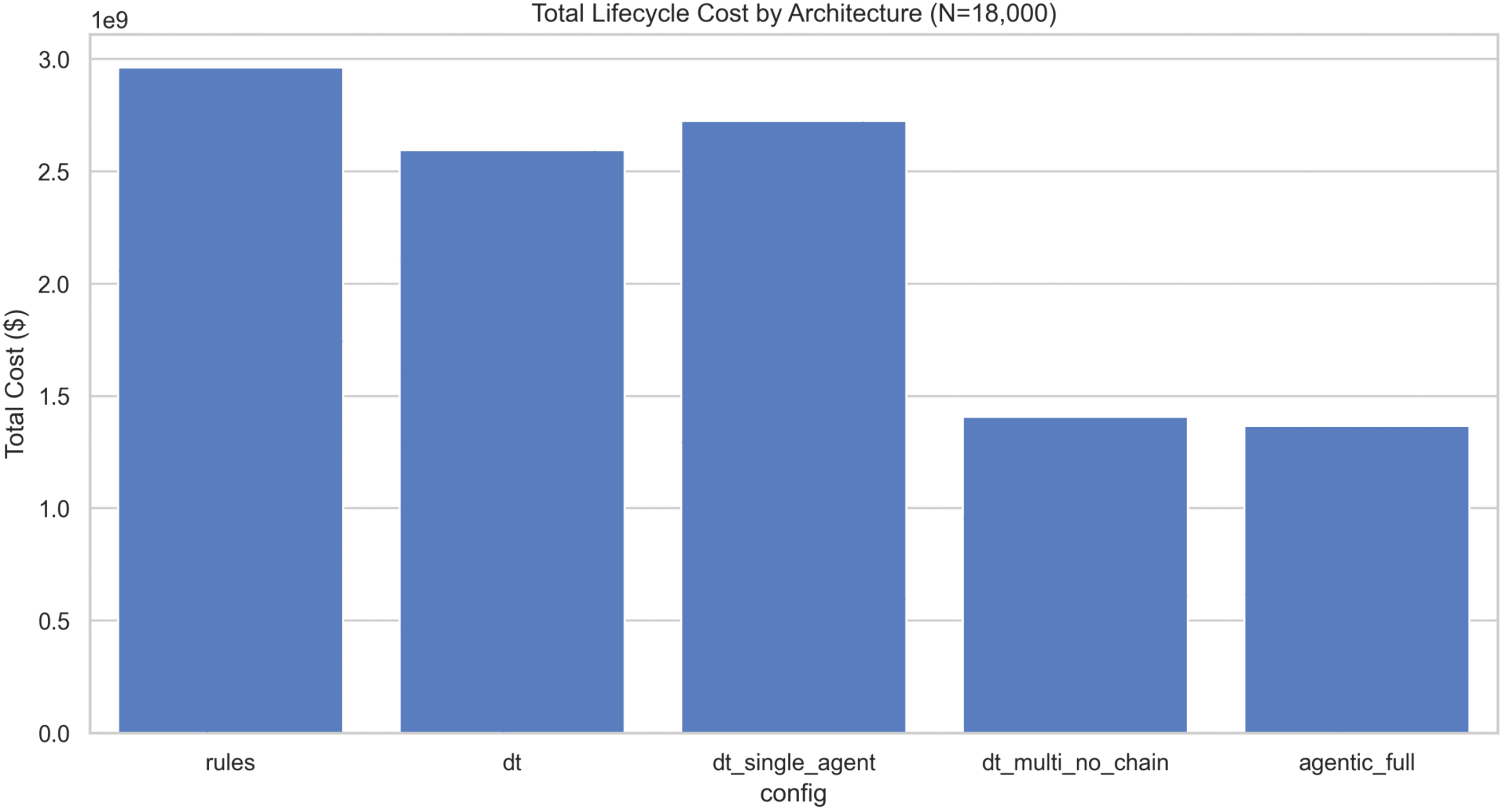
Estimated total lifecycle cost by architecture configuration (*N* = 18,000 incidents). Multi-agent orchestration is responsible for the dominant cost reduction; blockchain adds minimal overhead.

For each configuration, economic analysis calculations were made to estimate total costs over the lifecycle including incident costs, intervention costs, and missed mitigation penalties. Agentic Full came in with the lowest TLOC ($1.37B) compared to Rules-only ($2.96B) which is a 53.8% reduction. Even when not using blockchain, DT+Multi-Agent is able to achieve $1.41B, proving that blockchain does not impose significant cost overhead, but does provide a significant auditability benefit as well. Lifecycle cost comparison, shown in (Fig 10), shows the costs over the life of the configurations.

#### 6.6.4. Sensitivity analysis.

Spearman rank correlations were calculated to assess performance stability with respect to degradation parameter α and environmental noise σ. Both parameters showed strong negative monotonic associations with latency, in all configurations (all ρ ranging from −0.805 to −0.895 with all *p* < 0.001). Success rates showed moderate negative correlations (ρ between −0.431 and −0.518, all *p* < 0.001). Perhaps most importantly, the relative advantage of the various configurations did not depend on the parameters used, suggesting that the relative performance gains remain consistent over a range of stochastic conditions simulated in the parameter space. The reliability validation produced an accuracy of 75.9% for the classifiers, and a consistency delta (mean); train-test of 1.03 percentage points, which indicates internal consistency of the simulation framework.

### 6.7. Threats to validity and simulation limitations

We explicitly list threats to validity and limitations of this simulation study, in line with responsible reporting of synthetic results from evaluation studies.

**Internal validity – circular evaluation logic:** The simulation framework, configuration logic, and outcome metrics were all designed by the research team. The advantage is not just architectural, but the agentic configurations were crafted to take advantage of the services offered by DT simulation that are not available in the other configurations. We have attempted to ensure that each configuration’s design conforms to its architectural definition but have made no attempt to provide independent ground truth to support the mapping.

**Construct validity – latency-complexity inversion:** Higher complexity scenarios exhibit lower absolute latency across all configurations because the simulation encodes priority-escalation protocols that accelerate response routing for high-complexity incidents. This is not indicative of the complexity of the infrastructure in the real world, and more often than not is a function of how the simulation was designed rather than how it would operate in the real world, in which greater complexity often leads to more ambiguity in the diagnosis and longer to resolve. The relative advantage of multi-agent configurations remains the same across the different complexity levels, and the results for different complexity levels should not be viewed as representative of the real world complexity-response relationships.

**External validity – absence of real-world deployment:** The entire evaluation is synthetic. No real sensor reads were used, no real LLM API calls (with risk of hallucination, latency variation, token cost), no real blockchain deployment (throughput variations, consensus latency, infrastructure cost) and no real municipal API integrations. The simulation cannot be used to represent some of the things that can fail on the blockchain: in the simulation, LLM hallucination can result in unsafe mitigation plans; operational constraints of real municipal systems can be different; under adversarial prompts, agents can fail to coordinate. Results describe the framework’s simulated behaviour, and do not describe its actual behaviour in the real world.

**Statistical validity – pseudo-replication:** Although 18,000 incident records are analyzed, incidents within each run share the same random seed and simulation state trajectory, making them pseudo-replicates rather than independent observations. To overcome that, the primary run-level analysis (*N* = 30 independent seeds per configuration) is used instead of the primary seed-level analysis. All primary inferential claims are made based on run level statistics. The results are shown per incident and should not be regarded as being 3,600 independent results for each configuration.

**Adversarial robustness:** The simulation does not model LLM-specific adversarial conditions such as prompt injection attacks on the reasoning pipeline, agent policy drift under prolonged operation, or coordination failures under partial information. Resilience analysis (sensor spoofing) gives a partial description of a single class of threat, but is not a thorough adversarial analysis.

**Baseline optimization parity:** All baselines were configured with the same stochastic parameters and with no tuning that was specific to the configuration. The rules-only and the DT-only configurations have however simplified thresholds and models of operators respectively when compared to the multi-agent configurations that have a richer reasoning structure. This architectural asymmetry is a natural one inherent and not introduced by calibration and indicates that enhancements can’t be solely credited to the sophistication of the orchestration without real-world testing.

**Future validation requirements:** Translating these findings to operational practice requires real-world pilot studies incorporating: (i) actual sensor telemetry from civil infrastructure assets, (ii) real LLM API integration with hallucination monitoring, (iii) real permissioned blockchain deployment with throughput and latency measurement, (iv) actual municipal API integration with human approval workflows, and (v) longitudinal evaluation across seasonal and load variation cycles.

### 6.8. Interpretation

The simulation study shows that, in the controlled synthetic environment, multi-agent orchestration is a key factor in reducing latency, enhancing the probability of mitigation success, and reducing the workload for operators. The blockchain layer introduces decision audibility, with minimal performance impact. The single-agent reasoning and digital twin integration give modest, but not dominant contributions. The main scientific contribution of the evaluation is these component attributions, which have been set up by the ablation ladder.

The effect sizes observed (91.9% latency reduction, 66.2% relative success improvement) are related to the simulation design itself, and not to the extent of improvement that is expected in the real world. The run-level analysis, using *N* = 30 is appropriate for statistical inference, and the ranking of the configurations is fairly stable over the parameters varied in the simulated space. In order to have external validity, the pilot should be validated in a real world situation.

## 7. Conclusion

This paper introduced a conceptual framework for smart city civil infrastructure management using Agentic AI-Enhanced Digital Twin, which combines real-time DT state synchronization, orchestration of multi-agent Perception–Conceptualization–Action, and blockchain anchored provenance into an integrated operational design. The framework was tested with a controlled synthetic Monte Carlo simulation study, specifically as a characterization study, not an operational study, and with 18,000 incident simulations, five different architectural configurations and 30 independent runs.

The main finding of the evaluation is that orchestration by multiple agents is the biggest architectural factor contributing to simulated performance: 91.0% of the total latency reduction and 82.1% of total success improvement are due to the transition from a single-agent to multi-agent orchestration. The blockchain provenance layer provides mostly decision auditability, as measured by the fraction of justified decisions (91.7% vs. 0% baseline), while having only a small impact on operational metrics. The modest increases in each model (digital twin integration and single agent reasoning) are statistically not dominant.

The run-level primary analysis (*N* = 30 independent seeds per configuration) deals with issues of a nested data structure and the ranking of the configurations is stable across a range of different stochastic degradation and environmental noise parameters. Explicit threats-to-validity analysis recognizes circular evaluation logic, construct validity limitations coming from the latency-complexity inversion artifact, the fact that the work has never been deployed in the real world, and the possibility of pseudo-replication at the incident level as well as the lack of evaluation of the model against adversarial LLMs.

The findings offer simulation-level evidence of the effects that the incorporation of DT state synchronization along with structured multi-agent reasoning and blockchain provenance can offer in the context of the synthetic environment. For future work, real-world pilot deployments with real infrastructure telemetry, live deployment of LLM with hallucination monitoring, real deployment of blockchain applications and real integration of the municipal API is required to verify whether the simulated advantages in performance can be reproduced in practice.
