# Supplementary material for: Agentic AI-enhanced digital twins for Smart City civil infrastructure: A secure, autonomous and auditable management framework
Source: PLoS One. 2026 Jul 17;21(7):e0353610. doi: 10.1371/journal.pone.0353610 (PMC13379036; doi:10.1371/journal.pone.0353610)
Supplement: Attachment — Submitted filename: Response to reviewers.pdf [file pone.0353610.s002.pdf]

Response to Reviewers and Editor

Manuscript ID: PONE-D-26-02258

Title: Agentic AI-Enhanced Digital Twins for Smart City Civil Infrastructure: A Secure, Autonomous and Auditable Management Framework

Date: 04/03/2026

Dear Academic Editor,

We sincerely thank you and Reviewer #1 for the careful evaluation of our manuscript and for the constructive and technically rigorous feedback. We acknowledge that the original submission required substantial strengthening, particularly regarding experimental rigor, statistical validation, and data availability.

In response, we have comprehensively redesigned the experimental framework and substantially revised the manuscript. The revised version now presents a formally specified, statistically validated, and fully reproducible synthetic evaluation study comprising 10,800 incident observations across 30 independent simulation runs per configuration.

Below we provide a detailed point-by-point response.

Part I – Response to Journal Requirements

1. Manuscript Style and Formatting

Response:
The manuscript has been reformatted in strict accordance with the official PLOS ONE templates for title page and main body formatting.

Action Taken:

- Title page reformatted per PLOS ONE template.
- Section headings and references aligned with journal structure.
- All figure captions standardized.
- File naming conventions updated.
- Figures exported at publication-compliant resolution.

2. Code Sharing Policy

Response:
We acknowledge that the findings rely on author-generated simulation and analysis

code. To ensure full reproducibility and compliance with PLOS ONE's code-sharing policy, all source code has been made publicly available without restriction.

Action Taken:

- Complete synthetic simulation framework deposited.
- Statistical analysis scripts included.
- Configuration files and parameter specifications provided.
- Fixed global random seeds included for deterministic reproducibility.
- DOI-backed archival version created via Zenodo.

Repository Links:

https://doi.org/10.5281/zenodo.18849073
https://github.com/aliakarma/agentic-dt-study

3. Data Availability and Minimal Data Set

Response:
We acknowledge that the original submission did not provide the minimal dataset required for full replication. This has now been fully corrected.

The repository now includes the complete minimal dataset required to replicate all findings.

Publicly Available Materials:

- All 10,800 incident-level records
- Raw simulation outputs from 30 independent runs per configuration
- Values underlying all reported means and standard deviations
- Data used to generate all figures and tables
- Scenario complexity metadata
- Degradation and noise parameter values
- Statistical analysis outputs
- Environment configuration files

The revised Data Availability Statement explicitly references the DOI-backed archive and GitHub repository.

4. Figure 1 Copyright Compliance

Response:
The originally submitted Figure 1 contained elements incompatible with CC BY 4.0 licensing.

Action Taken:

Figure 1 has been completely redesigned as an original publication-grade system illustration. The new figure is fully CC BY 4.0 compliant and contains no third-party copyrighted content.

5. Figure Citation Corrections

Response:
All figures are now explicitly cited within the manuscript text at first mention. The omission noted by the journal has been corrected.

6. Financial Disclosure

A Financial Disclosure section has been added before the references in accordance with journal requirements.

Part II – Response to Reviewer #1

We sincerely appreciate Reviewer #1's detailed evaluation. The comments regarding technical soundness, statistical rigor, validation, and reproducibility were instrumental in guiding a substantial reconstruction of our experimental framework.

We acknowledge that the initial submission presented illustrative simulation results without formal replication, inferential testing, or open data transparency. The revised manuscript addresses these limitations comprehensively.

Comment 1: Technical Soundness

Reviewer Assessment: No

Response:
We agree that the original submission did not sufficiently formalize the experimental model. The revised manuscript now presents a fully specified synthetic evaluation framework.

Major Revisions Implemented:

1. Defined an explicit synthetic incident generation protocol.
2. Specified degradation and environmental noise parameterization.

3. Executed 30 independent simulation runs per configuration.
4. Evaluated 3,600 incidents per configuration (10,800 total).
5. Introduced factorial variation across three scenario complexity levels.
6. Restricted all claims strictly to statistically validated outcomes.

These revisions ensure that conclusions are supported by reproducible computational evidence.

Comment 2: Statistical Rigor

Reviewer Assessment: No

Response:
We agree that the initial statistical framework was insufficient. The revised manuscript now incorporates a comprehensive inferential validation protocol.

Statistical Enhancements Include:

- Reporting mean ± standard deviation for all continuous variables.
- Reporting 95% confidence intervals for all metrics.
- Shapiro–Wilk normality testing.
- Levene's homogeneity of variance testing.
- Welch's t-tests for unequal variances.
- Mann–Whitney U tests (non-parametric confirmation).
- Bonferroni correction for multiple comparisons.
- Effect size reporting (Cohen's d, rank-biserial r).
- Two-way factorial analysis using Aligned Rank Transform (ART).
- Logistic regression for binary mitigation success.
- Chi-squared tests with phi and Cramér's V effect sizes.
- A priori power analysis (G*Power 3.1.9.7).
- Sensitivity analysis using Spearman rank correlations across degradation and noise parameters.

These analyses are detailed in Section "Statistical Evaluation of the Agentic AI Framework."

The revised statistical framework substantially strengthens inferential validity and robustness.

Comment 3: Data Availability

Reviewer Assessment: No

Response:
This limitation has been fully resolved.

All minimal data required to replicate results are now publicly available, including raw incident-level records, parameter metadata, and full statistical scripts.

The manuscript now complies fully with the PLOS Data Policy.

Comment 4: Language and Clarity

Reviewer Assessment: Yes

Response:
We appreciate this assessment. Nevertheless, the manuscript underwent an additional editorial review to further improve clarity, precision, and consistency.

Comment 5: Novelty and State-of-the-Art Comparison

Reviewer Comment:

Further emphasize unique contributions and compare with state-of-the-art methods.

Response:
We have strengthened the manuscript accordingly.

Revisions Include:

1. Expanded Contributions subsection in the Introduction.
2. Added an explicit "Positioning Relative to State-of-the-Art" subsection.
3. Introduced a comparative feature table contrasting rule-based, DT-only, AI-assisted, and proposed architectures.
4. Clarified the architectural distinction of structured PCA orchestration.
5. Expanded explanation of blockchain-anchored provenance mechanisms.

These revisions clarify the novelty of integrating:

- Structured multi-agent orchestration,
- Digital twin simulation coupling,
- Governance-constrained mitigation synthesis,

- Cryptographically anchored auditability.

Concluding Statement

We believe the revised manuscript now addresses all concerns regarding technical soundness, statistical rigor, validation robustness, data availability, and novelty clarification.

The experimental evaluation has been reconstructed as a statistically validated, fully reproducible synthetic study supported by public data and code.

We sincerely thank the Editor and Reviewer #1 for their rigorous and constructive feedback, which has substantially improved the scientific quality and reproducibility of this work.

Sincerely,

Dr. Toqeer Ali Syed

Islamic University of Madinah
