# Supplementary material for: Agentic AI-enhanced digital twins for Smart City civil infrastructure: A secure, autonomous and auditable management framework
Source: PLoS One. 2026 Jul 17;21(7):e0353610. doi: 10.1371/journal.pone.0353610 (PMC13379036; doi:10.1371/journal.pone.0353610)
Supplement: Attachment — Submitted filename: Response_to_Reviewers_auresp_2.pdf [file pone.0353610.s003.pdf]

Response to Reviewers Manuscript: PONE-D-26-02258R1

Title: Agentic AI-Enhanced Digital Twins for Smart City Civil Infrastructure: A Secure, Autonomous and Auditable Management Framework

Date: 10, May 2026

We thank the Academic Editor and both Reviewers for the thorough and constructive evaluation of the revised manuscript. The reviewers acknowledge the substantial improvements in this revision and raise a focused set of methodological concerns regarding evaluation framing, implementation clarity, ablation studies, statistical validity, and limitations transparency. We have addressed each concern directly and substantively. Below we respond to every comment individually, describe all changes made, and reference the precise manuscript locations of each modification.

Summary of major changes in this revision:

- Expanded the evaluation from three to five architectural configurations, constituting a formal ablation ladder that isolates the contribution of each architectural component.
- Reframed the evaluation throughout as a *controlled synthetic Monte Carlo simulation study*, removing all language implying real-world operational validation.
- Adopted the simulation *run* (N = 30 per configuration) as the primary statistical unit, replacing incident-level inference and directly addressing pseudo-replication concerns.
- Added a dedicated **Threats to Validity and Simulation Limitations** subsection (Section 6.7) explicitly enumerating all ecological validity, construct validity, and statistical validity limitations.
- Revised Section 5 to clearly distinguish simulated, mocked, and conceptual components from any real implementations.
- Updated abstract, introduction, all results sections, and conclusion to reflect honest, scoped claims.
- Added four new figures (latency boxplot, success barplot, workload violin, attack detection, lifecycle cost, fatigue scatter).
- Added Table 3 presenting the formal ablation attribution analysis.

Response to Reviewer #2

Comment 2.1 – Circular Evaluation Logic

"This is the paper's fundamental weakness. The authors designed the simulator, designed the three configurations, and designed the outcome logic that determines all four metrics. The Agentic configuration wins because the simulation was built so that it would win. There is no independent ground truth, no external benchmark, and no adversarial conditions... The manuscript must be transparent about this: sophisticated inferential statistics cannot compensate for the absence of external validity when the entire data-generating process is author-controlled. The paper would be more honest -- and more publishable -- if it framed the evaluation as a Monte Carlo characterisation study rather than as hypothesis-testing evidence for operational superiority."

Response: We fully accept this critique and have restructured the evaluation framing accordingly throughout the manuscript. The reviewer is correct that framing author-controlled simulation results as evidence of real-world operational superiority is epistemologically inappropriate.

Changes Made:

- The **abstract** now explicitly states: *"This simulation study characterises framework behaviour under controlled stochastic conditions and does not constitute real-world operational validation."* All claims previously worded as demonstrations of "operational superiority" have been replaced with "simulated comparative performance".
- The **introduction** now frames the evaluation as a "controlled Monte Carlo simulation study" and lists as a contribution "an explicit characterisation of simulation assumptions, ecological validity limitations, and future real-world validation requirements."
- **Section 5** has been retitled "Simulation-Based Implementation Framework" and its opening paragraph states: *"We use the term simulation framework deliberately: the evaluation reported in Section 6 is conducted entirely within a controlled synthetic incident generation environment, not through deployment with real infrastructure, real municipal APIs, or real blockchain nodes."*
- A dedicated subsection parity and **(Section 6.7 Threats to Validity and Simulation Limitations)** enumerates six specific threats including circular evaluation logic, construct validity, external validity, statistical validity, adversarial

robustness, and baseline optimisation parity, and specifies five requirements for future real-world validation.

- The **conclusion** now states clearly: *"The framework was evaluated through a controlled synthetic Monte Carlo simulation study... explicitly framed as a characterisation study under controlled stochastic conditions rather than as real-world operational validation."* Operational superiority language has been removed throughout.
- The **interpretation** subsection (Section 6.6) states: *"The observed effect magnitudes... should be interpreted as characteristics of the simulation design rather than as projections of real-world improvement."*

Comment 2.2 – Actual Implementation Missing

"Section 5 describes a 7-layer prototype' in entirely aspirational terms. No evidence is presented that any of the following were implemented: a functioning Digital Twin with calibrated state estimation... a working LangChain/LangGraph pipeline... a permissioned blockchain node with measurable throughput, or integration with any municipal API. The paper oscillates between calling this a conceptualization framework' and a 'prototype'."

Response: The reviewer correctly identifies a critical ambiguity. We have resolved this by carefully auditing all language in Section 5 and adopting precise, consistent terminology distinguishing what was simulated, what was mocked, and what is conceptual.

Changes Made:

- **Section 5's title** has been changed from "Prototype Implementation" to "Simulation-Based Implementation Framework".
- Each layer subsection now carries a parenthetical qualifier: *(Simulated)*, *(Mocked)*, or *(Conceptual)* as appropriate, placed immediately in the subsection heading.
- **Layer 5 (Municipal Execution Systems)** now explicitly states: *"In the simulation framework, municipal execution systems... are represented as mocked API endpoints. Action payloads... are dispatched to these mocked endpoints, which return structured acknowledgements. The human-in-the-loop position is simulated as a probabilistic approval gate... Explicit human review of individual simulated incidents is not conducted."*
- **Layer 6 (Agentic AI Pipeline)** now clearly states: *"All configurations consume the same DT state and alert streams"* and distinguishes the five configurations by their simulated pipeline logic rather than by reference to deployed software.

- **Layer 7 (Blockchain)** now states: *"The blockchain audit module is implemented in the simulation as a hash-commit logging service... without deploying real blockchain infrastructure."*
- The words "prototype", "operational deployment", and "real-world implementation" have been removed or corrected throughout Section 5. The manuscript now consistently uses "simulation framework" and "conceptual architecture".

Comment 2.3 – No Ablation Studies

"Rules-only is architecturally incapable of cross-domain reasoning by definition. DT-only requires full human synthesis. Comparing the complete Agentic stack (DT + multi-agent reasoning + simulation + blockchain) against these stripped-down configurations tells the reader nothing about which component drives the improvement. The paper needs ablations: DT + single-agent (no multi-agent coordination), DT + multi-agent without blockchain, DT + rule-based automation without LLM reasoning."

Response: This is the most important new contribution of this revision. We have expanded the evaluation from three to five architectural configurations constituting a formal ablation ladder and report all results at the run level (N = 30 per configuration). The five configurations are: (1) Rules-only, (2) DT-only, (3) DT + Single-Agent, (4) DT + Multi-Agent without Blockchain, and (5) Agentic Full (DT + Multi-Agent + Blockchain). This directly instantiates the ablation structure the reviewer requested.

Changes Made:

- **Section 6.1 (Experimental Design)** now introduces all five configurations with precise architectural definitions and explicit baseline equivalence justification.
- **Table 2 (Overall Performance Summary)** now reports all five configurations across all four metrics with run-level means and 95% CIs.
- **Section 6.4.1 (Detection Latency)** reports pairwise ANOVA post-hoc comparisons for all adjacent ablation transitions, explicitly quantifying the marginal contribution of each component.
- **Section 6.4.2 (Mitigation Success)** and **Section 6.4.3 (Operator Workload)** follow the same pairwise ablation structure.
- **Section 6.5 (Ablation Analysis)** adds a dedicated ablation table (Table 3) summarising the marginal ΔLatency, ΔSuccess, and ΔJustified for each transition in the ablation ladder.

- The key ablation finding — that multi-agent orchestration (transition 3 → 4) accounts for 91.0% of total latency improvement and 82.1% of total success improvement, while blockchain (transition 4 → 5) contributes principally to auditability — is stated in the **abstract**, **introduction**, **results**, and **conclusion** as the primary scientific finding.
- New figures: latency_boxplot (Fig 5), success_rate_barplot (Fig 6), workload_violinplot (Fig 7), attack_detection_rate (Fig 8), fatigue_impact_scatter (Fig 9), lifecycle_cost_comparison (Fig 10) all show all five configurations.

Comment 2.4 – Statistical Methodology

"The 30-run replication design creates pseudo-replicates -- all runs use identical simulation logic... with only the random seed varying. This inflates the effective sample size without adding genuinely independent information. The >99% power to detect d ≥ 0.2 is trivially achieved when the experimenter controls both effect magnitude and noise floor. The ART interaction effect (configuration × complexity) exposes a construct validity problem: higher complexity yields lower latency because the complexity parameter encodes priority-escalation behavior rather than diagnostic difficulty... The logistic regression (OR = 2.48) is a bivariate restatement of the chi-squared contingency table."

Response: We agree with all three sub-points and have addressed each.

Changes Made:

- **Primary statistical unit changed to the run.** Section 6.1 now explicitly states: *"In recognition of the nested structure of the data — incidents are clustered within runs sharing the same random seed — the run is treated as the primary statistical unit. Run-level means aggregate 120 incidents per run to produce 30 observations per configuration, which are treated as approximately independent. Incident-level analyses characterise distributional properties but do not constitute independent observations."* All primary ANOVA, post-hoc, and effect-size calculations are now conducted on run-level means (N = 30).
- **The misleading power claim** (">99% power to detect d ≥ 0.2 at N = 3,600") has been removed and replaced with a run-level power statement: *"At effect sizes corresponding to observed differences, power exceeded 99% for all primary comparisons."* This is reported factually without implying that the large N is analytically independent.
- **The latency-complexity construct validity issue** is now explicitly acknowledged in Section 6.4.1 under a clearly labelled note and in the dedicated **Section 6.5 (Threats to Validity)**: *"Higher complexity scenarios exhibit lower absolute latency*

across all configurations because the simulation encodes priority-escalation protocols... This does not model real-world infrastructure behaviour." The relevant figure caption also notes this limitation.

- **The logistic regression** has been removed. The chi-squared tests are retained as the appropriate primary test for binary outcomes, with effect sizes reported. No redundant bivariate regression is included.
- **Distributional testing** is now reported at the run level (Shapiro-Wilk on N = 30 run means), where normality is not violated, correctly justifying the use of ANOVA. Incident-level non-normality is reported for characterisation purposes and explicitly noted as not affecting primary inference.

Comment 2.5 – Latency and Complexity Relationship

"The authors acknowledge that higher-complexity scenarios exhibit lower latency and attribute this to simulation design choices (aggressive escalation protocols). This concession undermines the simulation's ecological validity... its presence weakens confidence in all complexity-stratified results."

Response: We agree that this is a genuine ecological validity limitation and have treated it as such throughout the revision rather than as a footnote.

Changes Made:

- The latency-complexity inversion is now discussed in **Section 6.4.1**, **Section 6.7 (Threats to Validity)**, and the **conclusion**, with consistent language: *"This pattern reflects simulation design... does not model real-world infrastructure behaviour... complexity-stratified results should not be interpreted as characterising real-world complexity-response relationships."*
- The conclusion no longer relies on complexity-stratified results to support any primary claim.

Response to Reviewer #3

Comment 3.1 – Inferential Validity: Independence Assumption

"It is still not sufficiently clear whether the incident-level observations were treated as statistically independent when they are likely nested within simulation runs and scenario settings. If so, the effective sample size may be overstated... The authors should explicitly justify the independence assumption or preferably re-analyse the data at the run level or with an appropriate hierarchical/mixed-effects framework."

Response: We have re-analysed all primary results at the run level, as the reviewer recommends.

Changes Made:

- **Section 6.1** now explicitly establishes the run as the primary statistical unit, provides justification (runs differ by random seed, aggregating incidents within runs produces approximately independent summary statistics), and clearly distinguishes run-level inference from incident-level characterisation.
- All primary ANOVA, post-hoc, effect-size, and confidence interval calculations have been re-conducted on run-level means (N = 30). The revised Table 2 reports run-level statistics throughout.
- The phrase *"(incident-level characterisation; not independent observations)"* appears wherever incident-level figures or statistics are presented, preventing misinterpretation.
- The **Section 6.7 (Threats to Validity)** entry on "pseudo-replication" directly addresses this concern: *"Incidents within each run share the same random seed and simulation state trajectory, making them pseudo-replicates rather than independent observations. The primary run-level analysis (N = 30 independent seeds) addresses this... Incident-level results are presented for distributional characterisation only."*
- We note that a full hierarchical mixed-effects model would require incident-level covariates not retained in the current simulation output files. This is acknowledged as a limitation and identified as a methodological enhancement for future work with richer data retention.

Comment 3.2 – Scope of Conclusions

“Several statements in the title, abstract, and conclusion appear broader than the evidence currently supports. In particular, terms such as `secure' and broader operational claims should be stated more carefully unless they are directly validated. The conclusions should be limited to what has been demonstrated in the synthetic evaluation environment.”

Response: We have systematically audited and corrected all over-scoped claims throughout the manuscript.

Changes Made:

- The **abstract** now contains the explicit qualifier: *"These findings suggest that integrating simulation-enabled digital twins with governance-aware agentic orchestration can measurably enhance response efficiency... within the constraints of the synthetic evaluation environment."*
- The **conclusion** now states explicitly what was and was not demonstrated: *"These findings provide simulation-level evidence... Future work must include real-world pilot deployments... to determine whether the simulated performance advantages translate to operational practice."*
- The term "secure" in the title refers to the architectural design intention (blockchain-anchored provenance, permissioned access control), not an empirically validated security property. A footnote clarification has been added to Section 4.6 noting that the blockchain security properties are design-time properties of the permissioned ledger specification and are not empirically tested through adversarial attack in the evaluation.
- Phrases such as "demonstrates that", "proves that", and "validates that" have been replaced with "characterises", "provides simulation-level evidence that", and "suggests within the simulated environment".

Comment 3.3 – Component-Level Validation and Ablation

“The framework combines several elements -- digital twin synchronisation, agentic orchestration, and blockchain-based auditability -- but the evaluation does not yet fully isolate the contribution of each component. An ablation analysis, or at least a more explicit component-wise comparison, would substantially strengthen the paper.”

Response: See response to Reviewer #2, Comment 2.3 above. The full ablation analysis with five configurations now addresses this request directly. The key finding, that multi-

agent orchestration drives operational performance while blockchain drives auditability is the central quantitative contribution of the revised evaluation.

Changes Made: As itemised under Comment 2.3 above, including Table 3 (ablation attribution), revised Table 2 (all five configurations), and updated main results text throughout Section 6.

Comment 3.4 – Fairness of Baseline Comparison

"The fairness of the baseline comparison should be clarified further. The manuscript compares the proposed method with Rules-only and DT-only settings, but the exact degree of optimisation and tuning of these baselines should be made more explicit to ensure that the reported advantages are not partly due to asymmetry in implementation detail."

Response: We have added an explicit baseline equivalence statement and detailed each configuration's parameter choices.

Changes Made:

- **Section 6.1** now contains a dedicated **Baseline equivalence** paragraph: *"All five configurations used identical stochastic degradation parameters, environmental noise distributions, asset criticality weights, and scenario complexity proportions. No configuration received additional parameter tuning or optimisation beyond its architectural specification. The rules-only configuration applied identical detection thresholds across all runs; the DT-only configuration used a fixed operator synthesis latency and success probability function; the single-agent and multi-agent configurations used identical LLM simulation parameters. This design ensures that observed differences reflect architectural variation rather than asymmetric calibration."*
- Section 5's layer descriptions now specify the exact parameterisation of each simulated component, removing the ambiguity the reviewer identified.
- The **Threats to Validity** section (Section 6.7) acknowledges the residual limitation: *"The rules-only configuration's threshold logic and the DT-only configuration's operator synthesis model are necessarily simplified relative to the multi-agent configurations' rich reasoning structure. This architectural asymmetry is inherent to the comparison and is not an artifact of calibration, but it means that improvements cannot be attributed solely to orchestration sophistication without real-world validation."*

Comment 3.5 – Editorial Polish

"The manuscript is generally intelligible and written in good scientific English. Only minor editorial polishing is still needed, especially to reduce overstatement and improve the readability of some figures."

Response: We have conducted a full language review, removing overstatements, improving figure captions for readability, and ensuring consistent terminology (e.g., "simulated" vs. "prototype" vs. "conceptual") throughout.

Changes Made:

- All overstatement language removed as described in Comment 3.2 above.
- Figure captions revised for clarity and to include explicit notation of sample sizes and analysis level (run-level vs. incident-level).
- All five new figures include clear axis labels, legends, and captions specifying the data level.
- Notation consistency confirmed throughout (configuration labels, metric symbols, equation references).

Summary of All Modifications

1. **Abstract**: Fully revised to reflect Monte Carlo simulation study framing, five-configuration ablation, run-level statistics, updated performance figures, and scoped claims.
2. **Section 1 (Introduction)**: Softened deployment language; added simulation study framing; revised contribution list to include ablation and threat-of-validity contributions.
3. **Section 5 (Implementation)**: Retitled "Simulation-Based Implementation Framework"; all layers tagged as Simulated/Mocked/Conceptual; aspirational language removed; municipal API and blockchain layers clearly identified as mocked/simulated.
4. **Section 6.1 (Experimental Design)**: Expanded from 3 to 5 configurations; baseline equivalence paragraph added; primary statistical unit (run) established; power analysis updated.
5. **Section 6.2 (Metrics)**: Clarified run-level vs. incident-level reporting.
6. **Section 6.3 (Distributional Testing)**: Re-conducted at run level; normality satisfied; incident-level non-normality noted as characterisation only.
7. **Table 2**: Expanded to five configurations, run-level statistics.
8. **Section 6.4 (Primary Results)**: All pairwise comparisons conducted at run level with ANOVA + Tukey HSD; ablation transitions explicitly quantified; logistic regression removed.
9. **Table 3 (Ablation Analysis)**: New table showing marginal Δ for each architectural transition.
10. **Section 6.5 (Ablation Analysis)**: New subsection with formal attribution findings.
11. **Section 6.7 (Threats to Validity)**: New dedicated section with six explicit threats and five future validation requirements.
12. **Section 6.6 (Supplementary Analyses)**: Added resilience, workload-latency scatter, and lifecycle cost analyses with new figures.
13. **Section 7 (Conclusion)**: Fully revised to scope claims to simulation environment, highlight ablation as primary contribution, and specify future validation requirements.
14. **New Figures**: latency_boxplot (Fig 5), success_rate_barplot (Fig 6), workload_violinplot (Fig 7), attack_detection_rate (Fig 8), fatigue_impact_scatter (Fig 9), lifecycle_cost_comparison (Fig 10).
