# Supplementary material for: Agentic AI-enhanced digital twins for Smart City civil infrastructure: A secure, autonomous and auditable management framework
Source: PLoS One. 2026 Jul 17;21(7):e0353610. doi: 10.1371/journal.pone.0353610 (PMC13379036; doi:10.1371/journal.pone.0353610)
Supplement: Peer review history

# Peer review: Decision Letter 0

**DOI:** 10.1371/journal.pone.0353610.r001

**Authors:** Asaad Ahmed

24 Feb 2026

PONE-D-26-02258

Agentic AI-Enhanced Digital Twins for Smart City Civil Infrastructure: A Secure, Autonomous and Auditable Management Framework

PLOS One

Dear Dr. Akarma,

Thank you for submitting your manuscript to PLOS ONE. After careful consideration, we feel that it has merit but does not fully meet PLOS ONE’s publication criteria as it currently stands. Therefore, we invite you to submit a revised version of the manuscript that addresses the points raised during the review process.

==============================

Your manuscript has now been evaluated by an expert reviewer. After careful consideration of the review and my own assessment, I have concluded that the manuscript requires **Major Revision**  before it can be considered further for publication in PLOS ONE.

==============================

Please submit your revised manuscript by Apr 10 2026 11:59PM. If you will need more time than this to complete your revisions, please reply to this message or contact the journal office at plosone@plos.org. When you're ready to submit your revision, log on to https://www.editorialmanager.com/pone/ and select the 'Submissions Needing Revision' folder to locate your manuscript file.

Please include the following items when submitting your revised manuscript:

- A letter that responds to each point raised by the academic editor and reviewer(s). You should upload this letter as a separate file labeled 'Response to Reviewers'.
- A marked-up copy of your manuscript that highlights changes made to the original version. You should upload this as a separate file labeled 'Revised Manuscript with Track Changes'.
- An unmarked version of your revised paper without tracked changes. You should upload this as a separate file labeled 'Manuscript'.

If you would like to make changes to your financial disclosure, please include your updated statement in your cover letter. Guidelines for resubmitting your figure files are available below the reviewer comments at the end of this letter.

If applicable, we recommend that you deposit your laboratory protocols in protocols.io to enhance the reproducibility of your results. Protocols.io assigns your protocol its own identifier (DOI) so that it can be cited independently in the future. For instructions see: https://journals.plos.org/plosone/s/submission-guidelines#loc-laboratory-protocols. Additionally, PLOS ONE offers an option for publishing peer-reviewed Lab Protocol articles, which describe protocols hosted on protocols.io. Read more information on sharing protocols at https://plos.org/protocols?utm_medium=editorial-email&utm_source=authorletters&utm_campaign=protocols.

We look forward to receiving your revised manuscript.

Kind regards,

Asaad Ahmed Gad Elrab Ahmed

Academic Editor

PLOS One

Journal Requirements:

When submitting your revision, we need you to address these additional requirements.

1. Please ensure that your manuscript meets PLOS ONE's style requirements, including those for file naming. The PLOS ONE style templates can be found at

https://journals.plos.org/plosone/s/file?id=wjVg/PLOSOne_formatting_sample_main_body.pdf and

https://journals.plos.org/plosone/s/file?id=ba62/PLOSOne_formatting_sample_title_authors_affiliations.pdf

2.  Please note that PLOS One has specific guidelines on code sharing for submissions in which author-generated code underpins the findings in the manuscript. In these cases, we expect all author-generated code to be made available without restrictions upon publication of the work. Please review our guidelines at https://journals.plos.org/plosone/s/materials-and-software-sharing#loc-sharing-code and ensure that your code is shared in a way that follows best practice and facilitates reproducibility and reuse.

3. We note that your Data Availability Statement is currently as follows: All relevant data underlying the findings of this study are contained within the manuscript and its Supporting Information files. The experimental results reported in the paper are reproducible using the methods, configurations, and parameters described in the manuscript.

Please confirm at this time whether or not your submission contains all raw data required to replicate the results of your study. Authors must share the “minimal data set” for their submission. PLOS defines the minimal data set to consist of the data required to replicate all study findings reported in the article, as well as related metadata and methods (https://journals.plos.org/plosone/s/data-availability#loc-minimal-data-set-definition).

For example, authors should submit the following data:

- The values behind the means, standard deviations and other measures reported;

- The values used to build graphs;

- The points extracted from images for analysis.

Authors do not need to submit their entire data set if only a portion of the data was used in the reported study.

If your submission does not contain these data, please either upload them as Supporting Information files or deposit them to a stable, public repository and provide us with the relevant URLs, DOIs, or accession numbers. For a list of recommended repositories, please see https://journals.plos.org/plosone/s/recommended-repositories.

If there are ethical or legal restrictions on sharing a de-identified data set, please explain them in detail (e.g., data contain potentially sensitive information, data are owned by a third-party organization, etc.) and who has imposed them (e.g., an ethics committee). Please also provide contact information for a data access committee, ethics committee, or other institutional body to which data requests may be sent. If data are owned by a third party, please indicate how others may request data access.

4. We note that Figure 1 in your submission contain copyrighted images. All PLOS content is published under the Creative Commons Attribution License (CC BY 4.0), which means that the manuscript, images, and Supporting Information files will be freely available online, and any third party is permitted to access, download, copy, distribute, and use these materials in any way, even commercially, with proper attribution. For more information, see our copyright guidelines: http://journals.plos.org/plosone/s/licenses-and-copyright.

We require you to either (1) present written permission from the copyright holder to publish these figures specifically under the CC BY 4.0 license, or (2) remove the figures from your submission:

1. You may seek permission from the original copyright holder of Figure 1 to publish the content specifically under the CC BY 4.0 license.

We recommend that you contact the original copyright holder with the Content Permission Form (http://journals.plos.org/plosone/s/file?id=7c09/content-permission-form.pdf) and the following text:

“I request permission for the open-access journal PLOS ONE to publish XXX under the Creative Commons Attribution License (CCAL) CC BY 4.0 (http://creativecommons.org/licenses/by/4.0/). Please be aware that this license allows unrestricted use and distribution, even commercially, by third parties. Please reply and provide explicit written permission to publish XXX under a CC BY license and complete the attached form.”

Please upload the completed Content Permission Form or other proof of granted permissions as an "Other" file with your submission.

In the figure caption of the copyrighted figure, please include the following text: “Reprinted from [ref] under a CC BY license, with permission from [name of publisher], original copyright [original copyright year].”

2. If you are unable to obtain permission from the original copyright holder to publish these figures under the CC BY 4.0 license or if the copyright holder’s requirements are incompatible with the CC BY 4.0 license, please either i) remove the figure or ii) supply a replacement figure that complies with the CC BY 4.0 license. Please check copyright information on all replacement figures and update the figure caption with source information. If applicable, please specify in the figure caption text when a figure is similar but not identical to the original image and is therefore for illustrative purposes only.

5. Please ensure that you refer to Figure 5 in your text as, if accepted, production will need this reference to link the reader to the figure.

6. If the reviewer comments include a recommendation to cite specific previously published works, please review and evaluate these publications to determine whether they are relevant and should be cited. There is no requirement to cite these works unless the editor has indicated otherwise.

**Additional Editor Comments:**

Your manuscript has now been evaluated by an expert reviewer. After careful consideration of the review and my own assessment, I have concluded that the manuscript requires Major Revision before it can be considered further for publication in PLOS ONE.

[Note: HTML markup is below. Please do not edit.]

Reviewers' comments:

Reviewer's Responses to Questions

**Comments to the Author**

1. Is the manuscript technically sound, and do the data support the conclusions?

The manuscript must describe a technically sound piece of scientific research with data that supports the conclusions. Experiments must have been conducted rigorously, with appropriate controls, replication, and sample sizes. The conclusions must be drawn appropriately based on the data presented.

Reviewer #1: No

**********

2. Has the statistical analysis been performed appropriately and rigorously?

Reviewer #1: No

**********

3. Have the authors made all data underlying the findings in their manuscript fully available?

The PLOS Data policy requires authors to make all data underlying the findings described in their manuscript fully available without restriction, with rare exception (please refer to the Data Availability Statement in the manuscript PDF file). The data should be provided as part of the manuscript or its supporting information, or deposited to a public repository. For example, in addition to summary statistics, the data points behind means, medians and variance measures should be available. If there are restrictions on publicly sharing data—e.g. participant privacy or use of data from a third party—those must be specified.

Reviewer #1: No

**********

4. Is the manuscript presented in an intelligible fashion and written in standard English?

PLOS ONE does not copyedit accepted manuscripts, so the language in submitted articles must be clear, correct, and unambiguous. Any typographical or grammatical errors should be corrected at revision, so please note any specific errors here.

Reviewer #1: Yes

**********

5. Review Comments to the Author

Please use the space provided to explain your answers to the questions above. You may also include additional comments for the author, including concerns about dual publication, research ethics, or publication ethics. (Please upload your review as an attachment if it exceeds 20,000 characters)

Reviewer #1: I suggest that the authors further emphasize the unique contributions of their work. A more thorough comparison with current state of the art methods would significantly clarify the study's novelty. Additionally, enhancing the statistical framework perhaps by incorporating a sensitivity analysis or a formal validation set would increase the robustness of the findings. A more rigorous validation process is essential for the manuscript to meet the journal's publication standards.

**********

6. PLOS authors have the option to publish the peer review history of their article (what does this mean?). If published, this will include your full peer review and any attached files.

-->If you choose “no”, your identity will remain anonymous but your review may still be made public.

**Do you want your identity to be public for this peer review?**  For information about this choice, including consent withdrawal, please see our Privacy Policy.

Reviewer #1: No

**********

[NOTE: If reviewer comments were submitted as an attachment file, they will be attached to this email and accessible via the submission site. Please log into your account, locate the manuscript record, and check for the action link "View Attachments". If this link does not appear, there are no attachment files.]

To ensure your figures meet our technical requirements, please review our figure guidelines: https://journals.plos.org/plosone/s/figures

You may also use PLOS’s free figure tool, NAAS, to help you prepare publication quality figures: https://journals.plos.org/plosone/s/figures#loc-tools-for-figure-preparation.

NAAS will assess whether your figures meet our technical requirements by comparing each figure against our figure specifications.

# Author comment: Author response to Decision Letter 1

**DOI:** 10.1371/journal.pone.0353610.r002

6 Mar 2026

Response to Reviewers and Editor

Manuscript ID: PONE-D-26-02258

Title: Agentic AI-Enhanced Digital Twins for Smart City Civil Infrastructure: A Secure, Autonomous and Auditable Management Framework

Date: 04/03/2026

Dear Academic Editor,

We sincerely thank you and Reviewer #1 for the careful evaluation of our manuscript and for the constructive and technically rigorous feedback. We acknowledge that the original submission required substantial strengthening, particularly regarding experimental rigor, statistical validation, and data availability.

In response, we have comprehensively redesigned the experimental framework and substantially revised the manuscript. The revised version now presents a formally specified, statistically validated, and fully reproducible synthetic evaluation study comprising 10,800 incident observations across 30 independent simulation runs per configuration.

Below we provide a detailed point-by-point response.

Part I – Response to Journal Requirements

1. Manuscript Style and Formatting

Response:

The manuscript has been reformatted in strict accordance with the official PLOS ONE templates for title page and main body formatting.

Action Taken:

• Title page reformatted per PLOS ONE template.

• Section headings and references aligned with journal structure.

• All figure captions standardized.

• File naming conventions updated.

• Figures exported at publication-compliant resolution.

2. Code Sharing Policy

Response:

We acknowledge that the findings rely on author-generated simulation and analysis code. To ensure full reproducibility and compliance with PLOS ONE’s code-sharing policy, all source code has been made publicly available without restriction.

Action Taken:

• Complete synthetic simulation framework deposited.

• Statistical analysis scripts included.

• Configuration files and parameter specifications provided.

• Fixed global random seeds included for deterministic reproducibility.

• DOI-backed archival version created via Zenodo.

Repository Links:

https://doi.org/10.5281/zenodo.18849073

https://github.com/aliakarma/agentic-dt-study

3. Data Availability and Minimal Data Set

Response:

We acknowledge that the original submission did not provide the minimal dataset required for full replication. This has now been fully corrected.

The repository now includes the complete minimal dataset required to replicate all findings.

Publicly Available Materials:

• All 10,800 incident-level records

• Raw simulation outputs from 30 independent runs per configuration

• Values underlying all reported means and standard deviations

• Data used to generate all figures and tables

• Scenario complexity metadata

• Degradation and noise parameter values

• Statistical analysis outputs

• Environment configuration files

The revised Data Availability Statement explicitly references the DOI-backed archive and GitHub repository.

4. Figure 1 Copyright Compliance

Response:

The originally submitted Figure 1 contained elements incompatible with CC BY 4.0 licensing.

Action Taken:

Figure 1 has been completely redesigned as an original publication-grade system illustration. The new figure is fully CC BY 4.0 compliant and contains no third-party copyrighted content.

5. Figure Citation Corrections

Response:

All figures are now explicitly cited within the manuscript text at first mention. The omission noted by the journal has been corrected.

6. Financial Disclosure

A Financial Disclosure section has been added before the references in accordance with journal requirements.

Part II – Response to Reviewer #1

We sincerely appreciate Reviewer #1’s detailed evaluation. The comments regarding technical soundness, statistical rigor, validation, and reproducibility were instrumental in guiding a substantial reconstruction of our experimental framework.

We acknowledge that the initial submission presented illustrative simulation results without formal replication, inferential testing, or open data transparency. The revised manuscript addresses these limitations comprehensively.

Comment 1: Technical Soundness

Reviewer Assessment: No

Response:

We agree that the original submission did not sufficiently formalize the experimental model. The revised manuscript now presents a fully specified synthetic evaluation framework.

Major Revisions Implemented:

1. Defined an explicit synthetic incident generation protocol.

2. Specified degradation and environmental noise parameterization.

3. Executed 30 independent simulation runs per configuration.

4. Evaluated 3,600 incidents per configuration (10,800 total).

5. Introduced factorial variation across three scenario complexity levels.

6. Restricted all claims strictly to statistically validated outcomes.

These revisions ensure that conclusions are supported by reproducible computational evidence.

Comment 2: Statistical Rigor

Reviewer Assessment: No

Response:

We agree that the initial statistical framework was insufficient. The revised manuscript now incorporates a comprehensive inferential validation protocol.

Statistical Enhancements Include:

• Reporting mean ± standard deviation for all continuous variables.

• Reporting 95% confidence intervals for all metrics.

• Shapiro–Wilk normality testing.

• Levene’s homogeneity of variance testing.

• Welch’s t-tests for unequal variances.

• Mann–Whitney U tests (non-parametric confirmation).

• Bonferroni correction for multiple comparisons.

• Effect size reporting (Cohen’s d, rank-biserial r).

• Two-way factorial analysis using Aligned Rank Transform (ART).

• Logistic regression for binary mitigation success.

• Chi-squared tests with phi and Cramér’s V effect sizes.

• A priori power analysis (G*Power 3.1.9.7).

• Sensitivity analysis using Spearman rank correlations across degradation and noise parameters.

These analyses are detailed in Section “Statistical Evaluation of the Agentic AI Framework.”

The revised statistical framework substantially strengthens inferential validity and robustness.

Comment 3: Data Availability

Reviewer Assessment: No

Response:

This limitation has been fully resolved.

All minimal data required to replicate results are now publicly available, including raw incident-level records, parameter metadata, and full statistical scripts.

The manuscript now complies fully with the PLOS Data Policy.

Comment 4: Language and Clarity

Reviewer Assessment: Yes

Response:

We appreciate this assessment. Nevertheless, the manuscript underwent an additional editorial review to further improve clarity, precision, and consistency.

Comment 5: Novelty and State-of-the-Art Comparison

Reviewer Comment:

Further emphasize unique contributions and compare with state-of-the-art methods.

Response:

We have strengthened the manuscript accordingly.

Revisions Include:

1. Expanded Contributions subsection in the Introduction.

2. Added an explicit “Positioning Relative to State-of-the-Art” subsection.

3. Introduced a comparative feature table contrasting rule-based, DT-only, AI-assisted, and proposed architectures.

4. Clarified the architectural distinction of structured PCA orchestration.

5. Expanded explanation of blockchain-anchored provenance mechanisms.

These revisions clarify the novelty of integrating:

• Structured multi-agent orchestration,

• Digital twin simulation coupling,

• Governance-constrained mitigation synthesis,

• Cryptographically anchored auditability.

Concluding Statement

We believe the revised manuscript now addresses all concerns regarding technical soundness, statistical rigor, validation robustness, data availability, and novelty clarification.

The experimental evaluation has been reconstructed as a statistically validated, fully reproducible synthetic study supported by public data and code.

We sincerely thank the Editor and Reviewer #1 for their rigorous and constructive feedback, which has substantially improved the scientific quality and reproducibility of this work.

Sincerely,

Dr. Toqeer Ali Syed

Islamic University of Madinah

Attachment Submitted filename: Response to reviewers.pdf

# Peer review: Decision Letter 1

**DOI:** 10.1371/journal.pone.0353610.r003

**Authors:** Asaad Ahmed, Asaad Ahmed

6 May 2026

PONE-D-26-02258R1

Agentic AI-Enhanced Digital Twins for Smart City Civil Infrastructure: A Secure, Autonomous and Auditable Management Framework

PLOS One

Dear Dr. Akarma,

Thank you for submitting your manuscript to PLOS ONE. After careful consideration, we feel that it has merit but does not fully meet PLOS ONE’s publication criteria as it currently stands. Therefore, we invite you to submit a revised version of the manuscript that addresses the points raised during the review process.

Please submit your revised manuscript by Jun 20 2026 11:59PM. If you will need more time than this to complete your revisions, please reply to this message or contact the journal office at plosone@plos.org. When you're ready to submit your revision, log on to https://www.editorialmanager.com/pone/ and select the 'Submissions Needing Revision' folder to locate your manuscript file.

Please include the following items when submitting your revised manuscript:

- A letter that responds to each point raised by the academic editor and reviewer(s). You should upload this letter as a separate file labeled 'Response to Reviewers'.
- A marked-up copy of your manuscript that highlights changes made to the original version. You should upload this as a separate file labeled 'Revised Manuscript with Track Changes'.
- An unmarked version of your revised paper without tracked changes. You should upload this as a separate file labeled 'Manuscript'.

If you would like to make changes to your financial disclosure, please include your updated statement in your cover letter. Guidelines for resubmitting your figure files are available below the reviewer comments at the end of this letter.

If applicable, we recommend that you deposit your laboratory protocols in protocols.io to enhance the reproducibility of your results. Protocols.io assigns your protocol its own identifier (DOI) so that it can be cited independently in the future. For instructions see: https://journals.plos.org/plosone/s/submission-guidelines#loc-laboratory-protocols. Additionally, PLOS ONE offers an option for publishing peer-reviewed Lab Protocol articles, which describe protocols hosted on protocols.io. Read more information on sharing protocols at https://plos.org/protocols?utm_medium=editorial-email&utm_source=authorletters&utm_campaign=protocols.

As the corresponding author, your ORCID iD is verified in the submission system and will appear in the published article. PLOS supports the use of ORCID, and we encourage all coauthors to register for an ORCID iD and use it as well. Please encourage your coauthors to verify their ORCID iD within the submission system before final acceptance, as unverified ORCID iDs will not appear in the published article. *Only*  the individual author can complete the verification step; PLOS staff *cannot*  verify ORCID iDs on behalf of authors.

We look forward to receiving your revised manuscript.

Kind regards,

Asaad Ahmed Gad Elrab Ahmed

Academic Editor

PLOS One

Journal Requirements:

If the reviewer comments include a recommendation to cite specific previously published works, please review and evaluate these publications to determine whether they are relevant and should be cited. There is no requirement to cite these works unless the editor has indicated otherwise.

Additional Editor Comments :

Dear Authors,

Thank you for submitting the revised version of your manuscript, PONE-D-26-02258R1, entitled:

“Agentic AI-Enhanced Digital Twins for Smart City Civil Infrastructure: A Secure, Autonomous and Auditable Management Framework.”

We have now completed evaluation of your revised manuscript. The reviewers acknowledge that the revision represents a substantial improvement over the original submission, particularly with respect to statistical reporting, transparency, data/code availability, and positioning relative to prior work. The revised manuscript demonstrates significant effort and thoughtful engagement with the previous review comments.

However, after careful consideration of the reviewer reports and the revised manuscript, I have concluded that additional substantial revision is required before the manuscript can be considered further for publication.

[Note: HTML markup is below. Please do not edit.]

Reviewers' comments:

Reviewer's Responses to Questions

**Comments to the Author**

1. If the authors have adequately addressed your comments raised in a previous round of review and you feel that this manuscript is now acceptable for publication, you may indicate that here to bypass the “Comments to the Author” section, enter your conflict of interest statement in the “Confidential to Editor” section, and submit your "Accept" recommendation.

Reviewer #2: (No Response)

Reviewer #3: (No Response)

**********

2. Is the manuscript technically sound, and do the data support the conclusions?

The manuscript must describe a technically sound piece of scientific research with data that supports the conclusions. Experiments must have been conducted rigorously, with appropriate controls, replication, and sample sizes. The conclusions must be drawn appropriately based on the data presented.

Reviewer #2: No

Reviewer #3: No

**********

3. Has the statistical analysis been performed appropriately and rigorously?

Reviewer #2: No

Reviewer #3: No

**********

4. Have the authors made all data underlying the findings in their manuscript fully available?

The PLOS Data policy requires authors to make all data underlying the findings described in their manuscript fully available without restriction, with rare exception (please refer to the Data Availability Statement in the manuscript PDF file). The data should be provided as part of the manuscript or its supporting information, or deposited to a public repository. For example, in addition to summary statistics, the data points behind means, medians and variance measures should be available. If there are restrictions on publicly sharing data—e.g. participant privacy or use of data from a third party—those must be specified.

Reviewer #2: Yes

Reviewer #3: Yes

**********

5. Is the manuscript presented in an intelligible fashion and written in standard English?

PLOS ONE does not copyedit accepted manuscripts, so the language in submitted articles must be clear, correct, and unambiguous. Any typographical or grammatical errors should be corrected at revision, so please note any specific errors here.

Reviewer #2: Yes

Reviewer #3: Yes

**********

6. Review Comments to the Author

Please use the space provided to explain your answers to the questions above. You may also include additional comments for the author, including concerns about dual publication, research ethics, or publication ethics. (Please upload your review as an attachment if it exceeds 20,000 characters)

Reviewer #2: Summary

This paper proposes a 7-layer architecture combining Digital Twins, LangChain/LangGraph-based multi-agent orchestration (Perception–Conceptualization–Action), and permissioned blockchain auditability for smart city civil infrastructure management. Evaluation uses 10,800 synthetic incidents across three configurations and three complexity levels. The authors claim 45.5% latency reduction, 72.4% mitigation success (vs. 51.6% baseline), 71.9% workload reduction, and 91.9% blockchain-justified decisions.

Positives

The R1 revision represents a substantial and commendable effort. The statistical framework has been thoroughly upgraded — the authors now employ ART factorial analysis, Welch's t-tests with Mann-Whitney cross-validation, Bonferroni correction, effect size reporting (Cohen's d, Cramér's V), logistic regression, a priori power analysis, and Spearman sensitivity analysis. This is a well-constructed inferential toolkit. Data and code availability has been fully addressed through Zenodo (DOI-backed) and GitHub deposits, which is a meaningful improvement over the initial submission. The addition of a "Positioning Relative to State-of-the-Art" subsection with a comparative feature table (Table 1) clarifies the architectural novelty. The response to Reviewer #1 was thorough, professional, and transparent about the original submission's limitations.The core architectural idea — unifying DT state synchronization, structured multi-agent orchestration, and blockchain-anchored provenance — is conceptually interesting and addresses a genuine gap in civil infrastructure management.

Concerns

1. Circular Evaluation Logic: This is the paper's fundamental weakness. The authors designed the simulator, designed the three configurations, and designed the outcome logic that determines all four metrics. The Agentic configuration wins because the simulation was built so that it would win. There is no independent ground truth, no external benchmark, and no adversarial conditions (e.g., LLM hallucination producing unsafe mitigation plans, blockchain latency delaying time-critical decisions, agent coordination failures). The 10,800 incidents and 30 runs characterize the simulator's stochastic variance, not the framework's real-world viability.The manuscript must be transparent about this: sophisticated inferential statistics cannot compensate for the absence of external validity when the entire data-generating process is author-controlled. The paper would be more honest — and more publishable — if it framed the evaluation as a Monte Carlo characterization study rather than as hypothesis-testing evidence for operational superiority.

2. Actual Implementation Missing: Section 5 describes a 7-layer "prototype" in entirely aspirational terms. No evidence is presented that any of the following were implemented: a functioning Digital Twin with calibrated state estimation (the Kalman/particle filter equations are framed as "may be used"), a working LangChain/LangGraph pipeline that invokes an LLM and produces mitigation plans, a permissioned blockchain node with measurable throughput, or integration with any municipal API (real or mocked). The paper oscillates between calling this a "conceptualization framework" and a "prototype" — these carry very different epistemological commitments. Metrics reported with false precision (latency in seconds, workload in decisions/hour) are simulator outputs, not system measurements.

3. No Ablation Studies: Rules-only is architecturally incapable of cross-domain reasoning by definition. DT-only requires full human synthesis. Comparing the complete Agentic stack (DT + multi-agent reasoning + simulation + blockchain) against these stripped-down configurations tells the reader nothing about which component drives the improvement. The paper needs ablations: DT + single-agent (no multi-agent coordination), DT + multi-agent without blockchain, DT + rule-based automation without LLM reasoning. Without these, the claimed improvements cannot be attributed to any specific architectural contribution.

4. Statistical Methodology is lacking: The individual statistical methods are correctly chosen and executed. However, the 30-run replication design creates pseudo-replicates — all runs use identical simulation logic, configuration definitions, and parameter ranges with only the random seed varying. This inflates the effective sample size without adding genuinely independent information. The >99% power to detect d ≥ 0.2 is trivially achieved when the experimenter controls both effect magnitude and noise floor. The ART interaction effect (configuration × complexity) exposes a construct validity problem: higher complexity yields lower latency because the complexity parameter encodes priority-escalation behavior rather than diagnostic difficulty, confounding the factorial design. The logistic regression (OR = 2.48) is a bivariate restatement of the chi-squared contingency table and would be more informative if it included complexity, degradation, and noise as covariates.

5. Latency and Complexity Relationship is unclear: The authors acknowledge that higher-complexity scenarios exhibit lower latency and attribute this to simulation design choices (aggressive escalation protocols). This concession undermines the simulation's ecological validity — in real infrastructure operations, higher complexity generally implies greater diagnostic ambiguity and longer resolution. The authors correctly note this needs real-world confirmation, but its presence weakens confidence in all complexity-stratified results.

Overall - Revise and Resubmit.

Reviewer #3: Thank you for the substantial revisions made to this manuscript. The revised version is clearly improved in structure, transparency, statistical reporting, and data availability. In particular, the addition of a public data/code repository, the expanded statistical section, and the clearer positioning relative to prior work have strengthened the manuscript.

However, I do not yet consider the manuscript ready for acceptance.

My main remaining concern is the inferential validity of the statistical analysis. The revised manuscript reports 10,800 incident-level observations and 30 independent simulation runs per configuration, and applies multiple statistical tests. While this is a major improvement, it is still not sufficiently clear whether the incident-level observations were treated as statistically independent when they are likely nested within simulation runs and scenario settings. If so, the effective sample size may be overstated, which would affect p-values, confidence intervals, and effect-size interpretation. The authors should explicitly justify the independence assumption, or preferably re-analyze the data at the run level or with an appropriate hierarchical/mixed-effects framework.

My second concern is about the scope of the conclusions. The manuscript presents a synthetic simulation study, and the results support the claim that the proposed framework performs better than the selected baselines within that simulated setting. However, several statements in the title, abstract, and conclusion appear broader than the evidence currently supports. In particular, terms such as “secure” and broader operational claims should be stated more carefully unless they are directly validated. The conclusions should be limited to what has been demonstrated in the synthetic evaluation environment.

Third, the manuscript would benefit from stronger component-level validation. The framework combines several elements—digital twin synchronization, agentic orchestration, and blockchain-based auditability—but the evaluation does not yet fully isolate the contribution of each component. An ablation analysis, or at least a more explicit component-wise comparison, would substantially strengthen the paper.

Fourth, the fairness of the baseline comparison should be clarified further. The manuscript compares the proposed method with Rules-only and DT-only settings, but the exact degree of optimization and tuning of these baselines should be made more explicit to ensure that the reported advantages are not partly due to asymmetry in implementation detail.

Finally, the manuscript is generally intelligible and written in good scientific English. Only minor editorial polishing is still needed, especially to reduce overstatement and improve the readability of some figures.

In summary, the manuscript is promising and much improved, but I recommend major revision before it can be considered acceptable.

**********

7. PLOS authors have the option to publish the peer review history of their article (what does this mean?). If published, this will include your full peer review and any attached files.

If you choose “no”, your identity will remain anonymous but your review may still be made public.

**Do you want your identity to be public for this peer review?**  For information about this choice, including consent withdrawal, please see our Privacy Policy.

Reviewer #2: No

Reviewer #3: No

**********

[NOTE: If reviewer comments were submitted as an attachment file, they will be attached to this email and accessible via the submission site. Please log into your account, locate the manuscript record, and check for the action link "View Attachments". If this link does not appear, there are no attachment files.]

To ensure your figures meet our technical requirements, please review our figure guidelines: https://journals.plos.org/plosone/s/figures

You may also use PLOS’s free figure tool, NAAS, to help you prepare publication quality figures: https://journals.plos.org/plosone/s/figures#loc-tools-for-figure-preparation.

NAAS will assess whether your figures meet our technical requirements by comparing each figure against our figure specifications.

# Author comment: Author response to Decision Letter 2

**DOI:** 10.1371/journal.pone.0353610.r004

10 May 2026

We thank the Academic Editor and both Reviewers for the thorough and constructive evaluation of the revised manuscript. The reviewers acknowledge the substantial improvements in this revision and raise a focused set of methodological concerns regarding evaluation framing, implementation clarity, ablation studies, statistical validity, and limitations transparency. We have addressed each concern directly and substantively. Below we respond to every comment individually, describe all changes made, and reference the precise manuscript locations of each modification.

Summary of major changes in this revision:

• Expanded the evaluation from three to five architectural configurations, constituting a formal ablation ladder that isolates the contribution of each architectural component.

• Reframed the evaluation throughout as a controlled synthetic Monte Carlo simulation study, removing all language implying real-world operational validation.

• Adopted the simulation run (N = 30 per configuration) as the primary statistical unit, replacing incident-level inference and directly addressing pseudo-replication concerns.

• Added a dedicated Threats to Validity and Simulation Limitations subsection (Section 6.7) explicitly enumerating all ecological validity, construct validity, and statistical validity limitations.

• Revised Section 5 to clearly distinguish simulated, mocked, and conceptual components from any real implementations.

• Updated abstract, introduction, all results sections, and conclusion to reflect honest, scoped claims.

• Added four new figures (latency boxplot, success barplot, workload violin, attack detection, lifecycle cost, fatigue scatter).

• Added Table 3 presenting the formal ablation attribution analysis.

________________________________________

Response to Reviewer #2

________________________________________

Comment 2.1 – Circular Evaluation Logic

“This is the paper's fundamental weakness. The authors designed the simulator, designed the three configurations, and designed the outcome logic that determines all four metrics. The Agentic configuration wins because the simulation was built so that it would win. There is no independent ground truth, no external benchmark, and no adversarial conditions... The manuscript must be transparent about this: sophisticated inferential statistics cannot compensate for the absence of external validity when the entire data-generating process is author-controlled. The paper would be more honest -- and more publishable -- if it framed the evaluation as a Monte Carlo characterisation study rather than as hypothesis-testing evidence for operational superiority.”

Response: We fully accept this critique and have restructured the evaluation framing accordingly throughout the manuscript. The reviewer is correct that framing author-controlled simulation results as evidence of real-world operational superiority is epistemologically inappropriate.

Changes Made:

• The abstract now explicitly states: "This simulation study characterises framework behaviour under controlled stochastic conditions and does not constitute real-world operational validation." All claims previously worded as demonstrations of "operational superiority" have been replaced with "simulated comparative performance".

• The introduction now frames the evaluation as a "controlled Monte Carlo simulation study" and lists as a contribution "an explicit characterisation of simulation assumptions, ecological validity limitations, and future real-world validation requirements."

• Section 5 has been retitled "Simulation-Based Implementation Framework" and its opening paragraph states: "We use the term simulation framework deliberately: the evaluation reported in Section 6 is conducted entirely within a controlled synthetic incident generation environment, not through deployment with real infrastructure, real municipal APIs, or real blockchain nodes."

• A dedicated subsection parity and (Section 6.7 Threats to Validity and Simulation Limitations) enumerates six specific threats including circular evaluation logic, construct validity, external validity, statistical validity, adversarial robustness, and baseline optimisation parity, and specifies five requirements for future real-world validation.

• The conclusion now states clearly: "The framework was evaluated through a controlled synthetic Monte Carlo simulation study... explicitly framed as a characterisation study under controlled stochastic conditions rather than as real-world operational validation." Operational superiority language has been removed throughout.

• The interpretation subsection (Section 6.6) states: "The observed effect magnitudes... should be interpreted as characteristics of the simulation design rather than as projections of real-world improvement."

________________________________________

Comment 2.2 – Actual Implementation Missing

“Section 5 describes a 7-layer prototype' in entirely aspirational terms. No evidence is presented that any of the following were implemented: a functioning Digital Twin with calibrated state estimation... a working LangChain/LangGraph pipeline... a permissioned blockchain node with measurable throughput, or integration with any municipal API. The paper oscillates between calling this a conceptualization framework' and a ‘prototype’.”

Response: The reviewer correctly identifies a critical ambiguity. We have resolved this by carefully auditing all language in Section 5 and adopting precise, consistent terminology distinguishing what was simulated, what was mocked, and what is conceptual.

Changes Made:

• Section 5's title has been changed from "Prototype Implementation" to "Simulation-Based Implementation Framework".

• Each layer subsection now carries a parenthetical qualifier: (Simulated), (Mocked), or (Conceptual) as appropriate, placed immediately in the subsection heading.

• Layer 5 (Municipal Execution Systems) now explicitly states: "In the simulation framework, municipal execution systems... are represented as mocked API endpoints. Action payloads... are dispatched to these mocked endpoints, which return structured acknowledgements. The human-in-the-loop position is simulated as a probabilistic approval gate... Explicit human review of individual simulated incidents is not conducted."

• Layer 6 (Agentic AI Pipeline) now clearly states: "All configurations consume the same DT state and alert streams" and distinguishes the five configurations by their simulated pipeline logic rather than by reference to deployed software.

• Layer 7 (Blockchain) now states: "The blockchain audit module is implemented in the simulation as a hash-commit logging service... without deploying real blockchain infrastructure."

• The words "prototype", "operational deployment", and "real-world implementation" have been removed or corrected throughout Section 5. The manuscript now consistently uses "simulation framework" and "conceptual architecture".

________________________________________

Comment 2.3 – No Ablation Studies

“Rules-only is architecturally incapable of cross-domain reasoning by definition. DT-only requires full human synthesis. Comparing the complete Agentic stack (DT + multi-agent reasoning + simulation + blockchain) against these stripped-down configurations tells the reader nothing about which component drives the improvement. The paper needs ablations: DT + single-agent (no multi-agent coordination), DT + multi-agent without blockchain, DT + rule-based automation without LLM reasoning.”

Response: This is the most important new contribution of this revision. We have expanded the evaluation from three to five architectural configurations constituting a formal ablation ladder and report all results at the run level (N = 30 per configuration). The five configurations are: (1) Rules-only, (2) DT-only, (3) DT + Single-Agent, (4) DT + Multi-Agent without Blockchain, and (5) Agentic Full (DT + Multi-Agent + Blockchain). This directly instantiates the ablation structure the reviewer requested.

Changes Made:

• Section 6.1 (Experimental Design) now introduces all five configurations with precise architectural definitions and explicit baseline equivalence justification.

• Table 2 (Overall Performance Summary) now reports all five configurations across all four metrics with run-level means and 95% CIs.

• Section 6.4.1 (Detection Latency) reports pairwise ANOVA post-hoc comparisons for all adjacent ablation transitions, explicitly quantifying the marginal contribution of each component.

• Section 6.4.2 (Mitigation Success) and Section 6.4.3 (Operator Workload) follow the same pairwise ablation structure.

• Section 6.5 (Ablation Analysis) adds a dedicated ablation table (Table 3) summarising the marginal ΔLatency, ΔSuccess, and ΔJustified for each transition in the ablation ladder.

• The key ablation finding — that multi-agent orchestration (transition 3 → 4) accounts for 91.0% of total latency improvement and 82.1% of total success improvement, while blockchain (transition 4 → 5) contributes principally to auditability — is stated in the abstract, introduction, results, and conclusion as the primary scientific finding.

• New figures: latency_boxplot (Fig 5), success_rate_barplot (Fig 6), workload_violinplot (Fig 7), attack_detection_rate (Fig 8), fatigue_impact_scatter (Fig 9), lifecycle_cost_comparison (Fig 10) all show all five configurations.

________________________________________

Comment 2.4 – Statistical Methodology

“The 30-run replication design creates pseudo-replicates -- all runs use identical simulation logic... with only the random seed varying. This inflates the effective sample size without adding genuinely independent information. The >99% power to detect d ≥ 0.2 is trivially achieved when the experimenter controls both effect magnitude and noise floor. The ART interaction effect (configuration × complexity) exposes a construct validity problem: higher complexity yields lower latency because the complexity parameter encodes priority-escalation behavior rather than diagnostic difficulty... The logistic regression (OR = 2.48) is a bivariate restatement of the chi-squared contingency table.”

Response: We agree with all three sub-points and have addressed each.

Changes Made:

• Primary statistical unit changed to the run. Section 6.1 now explicitly states: "In recognition of the nested structure of the data — incidents are clustered within runs sharing the same random seed — the run is treated as the primary statistical unit. Run-level means aggregate 120 incidents per run to produce 30 observations per configuration, which are treated as approximately independent. Incident-level analyses characterise distributional properties but do not constitute independent observations." All primary ANOVA, post-hoc, and effect-size calculations are now conducted on run-level means (N = 30).

• The misleading power claim (">99% power to detect d ≥ 0.2 at N = 3,600") has been removed and replaced with a run-level power statement: "At effect sizes corresponding to observed differences, power exceeded 99% for all primary comparisons." This is reported factually without implying that the large N is analytically independent.

• The latency-complexity construct validity issue is now explicitly acknowledged in Section 6.4.1 under a clearly labelled note and in the dedicated Section 6.5 (Threats to Validity): "Higher complexity scenarios exhibit lower absolute latency across all configurations because the simulation encodes priority-escalation protocols... This does not model real-world infrastructure behaviour." The relevant figure caption also notes this limitation.

• The logistic regression has been removed. The chi-squared tests are retained as the appropriate primary test for binary outcomes, with effect sizes reported. No redundant bivariate regression is included.

• Distributional testing is now reported at the run level (Shapiro-Wilk on N = 30 run means), where normality is not violated, correctly justifying the use of ANOVA. Incident-level non-normality is reported for characterisation purposes and explicitly noted as not affecting primary inference.

________________________________________

Comment 2.5 – Latency and Complexity Relationship

“The authors acknowledge that higher-complexity scenarios exhibit lower latency and attribute this to simulation design choices (aggressive escalation protocols). This concession undermines the simulation's ecological validity... its presence weakens confidence in all complexity-stratified results.”

Response: We agree that this is a genuine ecological validity limitation and have treated it as such throughout the revision rather than as a footnote.

Changes Made:

• The latency-complexity inversion is now discussed in Section 6.4.1, Section 6.7 (Threats to Validity), and the conclusion, with consistent language: "This pattern reflects simulation design... does not model real-world infrastructure behaviour... complexity-stratified results should not be interpreted as characterising real-world complexity-response relationships."

• The conclusion no longer relies on complexity-stratified results to support any primary claim.

________________________________________

Response to Reviewer #3

________________________________________

Comment 3.1 – Inferential Validity: Independence Assumption

“It is still not sufficiently clear whether the incident-level observations were treated as statistically independent when they are likely nested within simulation runs and scenario settings. If so, the effective sample size may be overstated... The authors should explicitly justify the independence assumption or preferably re-analyse the data at the run level or with an appropriate hierarchical/mixed-effects framework.”

Response: We have re-analysed all primary results at the run level, as the reviewer recommends.

Changes Made:

• Section 6.1 now explicitly establishes the run as the primary statistical unit, provides justification (runs differ by random seed, aggregating incidents within runs produces approximately independent summary statistics), and clearly distinguishes run-level inference from incident-level characterisation.

• All primary ANOVA, post-hoc, effect-size, and confidence interval calculations have been re-conducted on run-level means (N = 30). The revised Table 2 reports run-level statistics throughout.

• The phrase "(incident-level characterisation; not independent observations)" appears wherever incident-level figures or statistics are presented, preventing misinterpretation.

• The Section 6.7 (Threats to Validity) entry on "pseudo-replication" directly addresses this concern: "Incidents within each run share the same random seed and simulation state trajectory, making them pseudo-replicates rather than independent observations. The primary run-level analysis (N = 30 independent seeds) addresses this... Incident-level results are presented for distributional characterisation only."

• We note that a full hierarchical mixed-effects model would require incident-level covariates not retained in the current simulation output files. This is acknowledged as a limitation and identified as a methodological enhancement for future work with richer data retention.

________________________________________

Comment 3.2 – Scope of Conclusions

“Several statements in the title, abstract, and conclusion appear broader than the evidence currently supports. In particular, terms such as `secure' and broader operational claims should be stated more carefully unless they are directly validated. The conclusions should be limited to what has been demonstrated in the synthetic evaluation environment.”

Response: We have systematically audited and corrected all over-scoped claims throughout the manuscript.

Changes Made:

• The abstract now contains the explicit qualifier: "These findings suggest that integrating simulation-enabled digital twins with governance-aware agentic orchestration can measurably enhance response efficiency... within the constraints of the synthetic evaluation environment."

• The conclusion now states explicitly what was and was not demonstrated: "These findings provide simulation-level evidence... Future work must include real-world pilot deployments... to determine whether th

Attachment Submitted filename: Response_to_Reviewers_auresp_2.pdf

# Peer review: Decision Letter 2

**DOI:** 10.1371/journal.pone.0353610.r005

**Authors:** Asaad Ahmed, Asaad Ahmed, Asaad Ahmed

25 Jun 2026

Agentic AI-Enhanced Digital Twins for Smart City Civil Infrastructure: A Secure, Autonomous and Auditable Management Framework

PONE-D-26-02258R2

Dear Dr. Ali Akarma,

We’re pleased to inform you that your manuscript has been judged scientifically suitable for publication and will be formally accepted for publication once it meets all outstanding technical requirements.

Within one week, you’ll receive an e-mail detailing the required amendments. When these have been addressed, you’ll receive a formal acceptance letter and your manuscript will be scheduled for publication.

An invoice will be generated when your article is formally accepted. Please note, if your institution has a publishing partnership with PLOS and your article meets the relevant criteria, all or part of your publication costs will be covered. Please make sure your user information is up-to-date by logging into Editorial Manager at Editorial Manager® and clicking the ‘Update My Information' link at the top of the page. For questions related to billing, please contact billing support.

If your institution or institutions have a press office, please notify them about your upcoming paper to help maximize its impact. If they’ll be preparing press materials, please inform our press team as soon as possible -- no later than 48 hours after receiving the formal acceptance. Your manuscript will remain under strict press embargo until 2 pm Eastern Time on the date of publication. For more information, please contact onepress@plos.org.

Kind regards,

Asaad Ahmed Gad Elrab Ahmed

Academic Editor

PLOS One

Additional Editor Comments (optional):

Reviewers' comments:

Reviewer's Responses to Questions

**Comments to the Author**

1. If the authors have adequately addressed your comments raised in a previous round of review and you feel that this manuscript is now acceptable for publication, you may indicate that here to bypass the “Comments to the Author” section, enter your conflict of interest statement in the “Confidential to Editor” section, and submit your "Accept" recommendation.

Reviewer #4: All comments have been addressed

**********

2. Is the manuscript technically sound, and do the data support the conclusions?

The manuscript must describe a technically sound piece of scientific research with data that supports the conclusions. Experiments must have been conducted rigorously, with appropriate controls, replication, and sample sizes. The conclusions must be drawn appropriately based on the data presented.

Reviewer #4: Yes

**********

3. Has the statistical analysis been performed appropriately and rigorously?

Reviewer #4: Yes

**********

4. Have the authors made all data underlying the findings in their manuscript fully available?

The PLOS Data policy requires authors to make all data underlying the findings described in their manuscript fully available without restriction, with rare exception (please refer to the Data Availability Statement in the manuscript PDF file). The data should be provided as part of the manuscript or its supporting information, or deposited to a public repository. For example, in addition to summary statistics, the data points behind means, medians and variance measures should be available. If there are restrictions on publicly sharing data—e.g. participant privacy or use of data from a third party—those must be specified.

Reviewer #4: Yes

**********

5. Is the manuscript presented in an intelligible fashion and written in standard English?

PLOS ONE does not copyedit accepted manuscripts, so the language in submitted articles must be clear, correct, and unambiguous. Any typographical or grammatical errors should be corrected at revision, so please note any specific errors here.

Reviewer #4: Yes

**********

6. Review Comments to the Author

Please use the space provided to explain your answers to the questions above. You may also include additional comments for the author, including concerns about dual publication, research ethics, or publication ethics. (Please upload your review as an attachment if it exceeds 20,000 characters)

Reviewer #4: The authors have done an exemplary job addressing the methodological and framing concerns raised during the previous round of review. The manuscript is vastly improved and now represents a rigorous, transparent, and valuable contribution to the field of smart city infrastructure management.

Specifically, the following revisions are highly commended:

Ablation Study: Expanding the evaluation from three to five configurations was a crucial step. It successfully isolates the marginal contributions of the multi-agent orchestration versus the blockchain layer, which significantly strengthens the paper's core claims.

Statistical Rigor: Shifting the primary statistical unit from the incident level to the run level (N=30) was exactly the right move to correct the pseudo-replication issue. The accompanying power analysis and normality checks justify the use of ANOVA and make the inferential statistics structurally sound.

Transparent Framing: Reframing the paper as a "controlled synthetic Monte Carlo simulation study" rather than a real-world operational validation demonstrates excellent scientific integrity. The explicit distinction between simulated, mocked, and conceptual components in Section 5 adds much-needed clarity.

Limitations: The new "Threats to Validity and Simulation Limitations" section (6.7) is thorough and honest. Addressing the latency-complexity inversion as a construct validity artifact of the simulation logic, rather than hiding it, builds immense trust with the reader.

The language has been thoroughly polished to remove overstatements, and the new visualizations (particularly the workload violin plots and the latency boxplots) effectively communicate the updated findings. The authors have met the high standards required for publication.

**********

7. PLOS authors have the option to publish the peer review history of their article (what does this mean?). If published, this will include your full peer review and any attached files.

If you choose “no”, your identity will remain anonymous but your review may still be made public.

**Do you want your identity to be public for this peer review?**  For information about this choice, including consent withdrawal, please see our Privacy Policy.

Reviewer #4: No

**********

# Editor report: Acceptance letter

**DOI:** 10.1371/journal.pone.0353610.r006

**Authors:** Asaad Ahmed, Asaad Ahmed, Asaad Ahmed

PONE-D-26-02258R2

PLOS One

Dear Dr. Akarma,

I'm pleased to inform you that your manuscript has been deemed suitable for publication in PLOS One. Congratulations! Your manuscript is now being handed over to our production team.

At this stage, our production department will prepare your paper for publication. This includes ensuring the following:

* All references, tables, and figures are properly cited

* All relevant supporting information is included in the manuscript submission,

* There are no issues that prevent the paper from being properly typeset

You will receive further instructions from the production team, including instructions on how to review your proof when it is ready. Please keep in mind that we are working through a large volume of accepted articles, so please give us a few days to review your paper and let you know the next and final steps.

Lastly, if your institution or institutions have a press office, please let them know about your upcoming paper now to help maximize its impact. If they'll be preparing press materials, please inform our press team within the next 48 hours. Your manuscript will remain under strict press embargo until 2 pm Eastern Time on the date of publication. For more information, please contact onepress@plos.org.

You will receive an invoice from PLOS for your publication fee after your manuscript has reached the completed accept phase. If you receive an email requesting payment before acceptance or for any other service, this may be a phishing scheme. Learn how to identify phishing emails and protect your accounts at https://explore.plos.org/phishing.

If we can help with anything else, please email us at customercare@plos.org.

Thank you for submitting your work to PLOS ONE and supporting open access.

Kind regards,

PLOS ONE Editorial Office Staff

on behalf of

Professor Asaad Ahmed Gad Elrab Ahmed

Academic Editor

PLOS One
